# COVID-19 Vaccines Confer Protection in Hospitalized Pregnant and Postpartum Women with Severe COVID-19: A Retrospective Cohort Study

**DOI:** 10.3390/vaccines10050749

**Published:** 2022-05-10

**Authors:** Cristiane de Freitas Paganoti, Rafaela Alkmin da Costa, Aris T. Papageorghiou, Fabrício da Silva Costa, Silvana Maria Quintana, Luciana Graziela de Godoi, Nátaly Adriana Jiménez Monroy, Agatha Sacramento Rodrigues, Rossana Pulcineli Vieira Francisco

**Affiliations:** 1 Divisão de Clinica Obstetrica, Hospital das Clinicas HCFMUSP, Faculdade de Medicina da Universidade de São Paulo, 255 Dr Eneas Carvalho de Aguiar Avenue, 10th floor, São Paulo 05403-000, Brazil; rafaela.alkmin@hc.fm.usp.br; 2 Nuffield Department of Women’s and Reproductive Health, University of Oxford, Women’s Centre, John Radcliffe Hospital, Headington, Oxford OX3 9DU, UK; aris.papageorghiou@wrh.ox.ac.uk; 3 Maternal Fetal Medicine Unit, Gold Coast University Hospital, School of Medicine, Griffith University, Gold Coast 4222, Australia; fabricio.dasilvacosta@health.qld.gov.au; 4 Gynecology and Obstetric Department, Ribeirão Preto Medical School, University of São Paulo, 3900 Bandeirantes Avenue, Ribeirão Preto, São Paulo 14049-900, Brazil; quintana@fmrp.usp.br; 5 Departamento de Estatística, Universidade Federal do Espírito Santo, 514 Fernando Ferrari Avenue, Goiabeira, Vitória 29075-910, Brazil; lgodoi.cce.ufes@gmail.com (L.G.d.G.); nataly.monroy@ufes.br (N.A.J.M.); agatha.rodrigues@ufes.br (A.S.R.); 6 Disciplina de Obstetrícia, Departamento de Obstetrícia e Ginecologia, Hospital das Clinicas HCFMUSP, Faculdade de Medicina da Universidade de São Paulo, São Paulo 05508-070, Brazil; rossana.francisco@hc.fm.usp.br

**Keywords:** COVID-19 vaccines, pregnancy, maternal mortality, intubation, intensive care unit, severe acute respiratory syndrome

## Abstract

The coronavirus disease 2019 (COVID-19) pandemic has had deleterious effects among the obstetric population. Pregnant and postpartum women constitute a high-risk group for severe COVID-19. Vaccination reduces the risk of infection, but it is not known whether women who become infected despite vaccination have a milder course of disease than those who had not been vaccinated. This retrospective cohort study evaluated whether vaccination reduces the severity of COVID-19 infection, as measured by severe maternal morbidity and mortality among hospitalized pregnant and postpartum individuals. A total of 2284 pregnant and postpartum women hospitalized with severe COVID-19 were included. Those who did and who did not receive COVID-19 vaccination were compared. The rates of intensive care unit admission, intubation, and mortality were significantly lower among subjects in the vaccinated group (*p* < 0.001, *p* < 0.001 and *p* < 0.001, respectively). The numbers of patients who needed to be vaccinated to avoid one case of intensive care unit admission, intubation, or death due to COVID-19 were 7, 7, and 9, respectively. The COVID-19 vaccine offers protective effects against intensive care unit admission, intubation, and death in hospitalized pregnant and postpartum women with severe SARS-CoV-2-induced SARS.

## 1. Introduction

The coronavirus disease 2019 (COVID-19) pandemic has had deleterious effects among the obstetric population [1,2,3,4,5], especially in low- and middle-income countries. In Brazil, a total of 1511 maternal deaths were documented in 2021, corresponding to a 329% increase from figures in 2020 [6,7].

The first randomized clinical trials (RCTs) examining the effectiveness and safety of COVID-19 vaccines did not include pregnant and postpartum women [8,9,10]. This policy was widely discussed [8,11,12], but regardless of reasons, the main findings are that, at least in the early stages of vaccine dissemination, there was little information to guide prospective parents on the effects and safety of vaccines in pregnancy. Thus, although they are considered to be a high-risk group for severe COVID-19, such concerns resulted in low vaccine uptake amongst pregnant women. This continued even after international and national guidance changed to include pregnant individuals in vaccination programs [9,13].

Considering the serious maternal and perinatal morbidity, and secondary mortality to COVID-19, most countries started vaccinating pregnant and postpartum women [14,15,16]. This decision was primarily based on published evidence of the first observational studies and case series of individuals who became pregnant after receiving the vaccine or received it during pregnancy [10,17,18,19,20,21]. In addition to such direct evidence, safety data on the administration of inactivated-virus vaccines during pregnancy (such as H1N1 and Tdap) [22,23] have allowed for the vaccination of pregnant and postpartum women with inactivated virus and messenger RNA vaccines.

Vaccination in pregnancy is clearly a key preventive measure for a decrease in the likelihood of the disease [8,24,25]. In this study, we evaluate whether vaccination also reduces the severity of COVID-19 infection, as measured by maternal adverse outcomes including mortality among hospitalized pregnant and postpartum individuals.

## 2. Materials and Methods

This is a retrospective cohort study (STROBE checklist—supplemental material) based on data collected by the Influenza Epidemiological Surveillance Information System (Sistema de Informação de Vigilância Epidemiológica da Gripe; SIVEP-Gripe). The SIVEP-Gripe database (available at https://opendatasus.saude.gov.br/, accessed on 5 April 2022), records demographic, clinical, and epidemiological data, and laboratory and etiological results. There is also information on hospital admission and disease progression (recovery or death).

This nationwide surveillance database was created in 2000 to monitor severe acute respiratory infections and data on viral circulation and respiratory infections in Brazil. Individuals presenting with fever, cough, and/or sore throat in sentinel monitoring units were documented in the database. In view of the H1N1 pandemic in 2009, the rigorous surveillance of individuals with severe acute respiratory syndrome (SARS), defined as the presence of fever, cough, and dyspnea, was adopted. This includes compulsory notification of all SARS cases. Since 2010, only hospitalized cases of SARS in both public and private hospitals, and cases of deaths caused by SARS, irrespective of hospitalization status, have been reported.

As viral surveillance for public health purposes has dynamic characteristics, there are frequent updates in notification guidelines. Since the start of the COVID-19 pandemic, cases of SARS with the presence of at least two of the following symptoms have been reported: fever, chills, sore throat, headache, cough, runny nose, and olfactory or taste disorders, plus dyspnea or chest pressure or saturation less than 95% or blue coloration of lips or face.

In this study, we evaluate clinical and epidemiological features, and clinical outcomes of pregnant and postpartum women hospitalized with severe SARS-CoV-2, comparing those who received COVID-19 vaccination with those who did not receive it. Primary outcomes were admission to an intensive care unit, the need for invasive ventilation, and death. Data were obtained from women enrolled in the study between 2 May 2021 and 27 November 2021.

Patients were selected according to the following inclusion criteria: pregnant and postpartum women of childbearing age (10–55 years old), COVID-19 disease confirmed by positive reverse transcription–polymerase chain reaction (RT-PCR) SARS-CoV-2 or antigen, the exclusion of influenza infection by negative RT-PCR or antigen for influenza, availability of the outcome (recovery or death), and the reliability of vaccination status. Since this was a retrospective study, no exclusion criteria were determined.

Considering the retrospective nature of the study and the anonymity of SIVEP-GRIPE data, no prior informed consent or approval by the institutional ethics committee was necessary, which is in agreement with the relevant Brazilian ethics in terms of research resolution [26].

### Statistical Analyses

Enrolled patients were grouped according to their COVID-19 vaccination status, namely, vaccinated or unvaccinated. Only women with two registered vaccine doses or no vaccine doses were included in the analysis.

Categorical variables are presented as the absolute and relative frequencies, and compared using the chi-squared test or Fisher’s exact test, as applicable. The odds ratio (OR) and 95% confidence interval (CI 95%) were considered to be measures of association. Continuous variables are presented as the mean and standard deviation (SD) and compared using the Student’s *t*-test. A *p*-value of <0.05 was considered to be statistically significant.

Propensity score matching (PSM) was used to estimate and balance the groups in relation to confounding variables. The propensity score was estimated using logistic regression, and 1:1 matching was performed by considering nearest-neighbor matching [27]. The average treatment effect (ATT) was estimated for the vaccinated group effect, and the control variables considered were age and preexisting cardiac disease. PSM results and standardized difference after matching are shown in the supplemental material.

All analyses were performed using R (R Foundation for Statistical Computing Platform, version 4.0.3) [28], and PSM was carried out with the R Matchlt package [29].

Only valid responses for each variable were considered, with the percentage of missing data depending on the variable in question. The valid number of cases for each variable was stated in the tables. Missing data received no treatment and were not considered for analysis.

The number needed to treat (NNT) was calculated, and cost values for the COVID-19 vaccine [30] and intensive care unit (ICU) daily rate [31] under the Brazilian health system were estimated.

## 3. Results

### 3.1. Study Population

**Figure 1:**
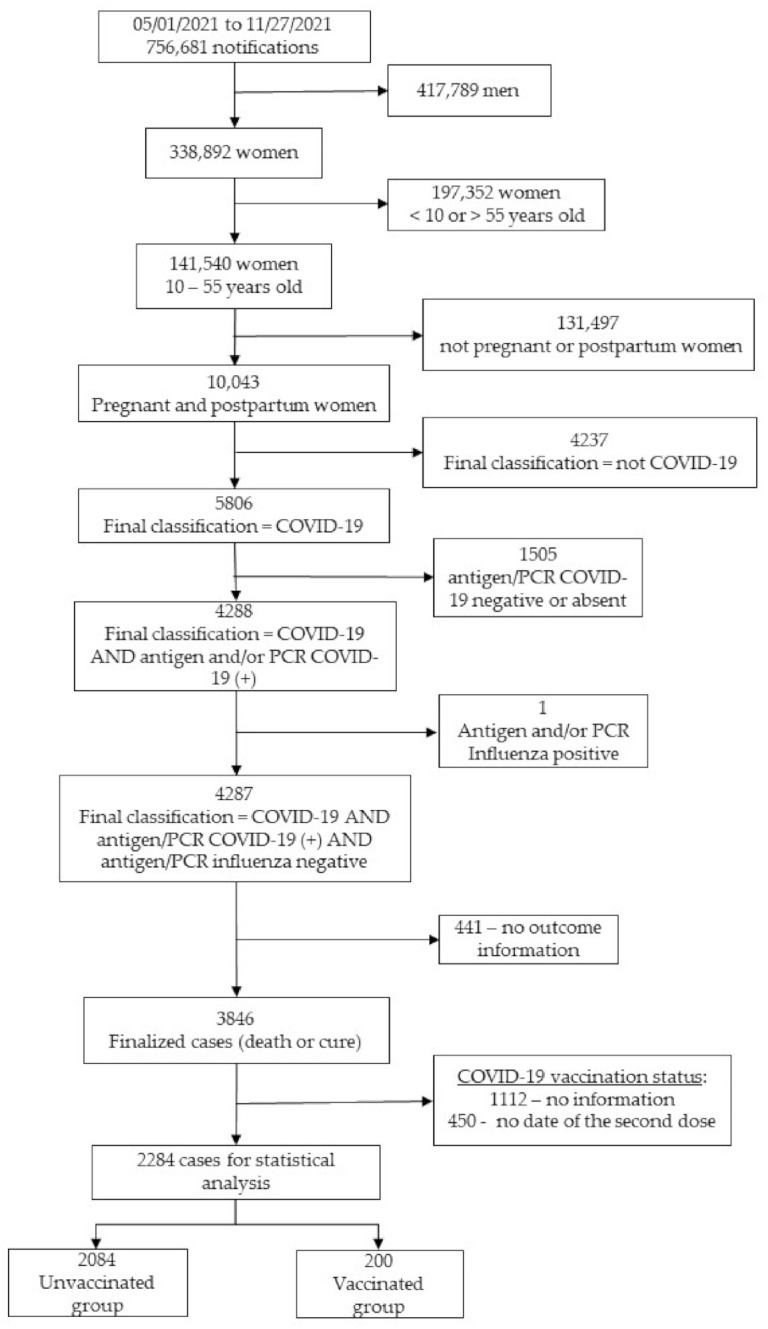
Participant flowchart. COVD-19, coronavirus disease 2019; PCR—polymerase chain reaction.

A total of 2284 patients were analyzed (Figure 1) and divided into two groups according to their COVID-19 vaccination status: unvaccinated (*n* = 2084; 91.2%) and vaccinated (*n* = 200; 8.8%).

### 3.2. Baseline Characteristics of Enrolled Subjects

The sociodemographic features of the subjects are presented in Table 1. Women in the unvaccinated group were younger than those in the vaccinated group (*p* = 0.003); however, the effect was small (d-Cohen coefficient = −0.24 (95% CI −0.39; −0.10)). There were no statistical differences between groups in terms of ethnicity or education level (*p* = 0.116).

Cardiac disease and immunosuppression were the only comorbidities showing statistical difference between groups; as expected, these were less frequent among women in the unvaccinated group compared to those in the vaccinated group (12.4% vs. 23.7%; *p* = 0.004 and 1.4% vs. 5.6%; *p* = 0.015, respectively). Both conditions increased the chance of having been vaccinated by 2.20 (95% CI 1.31–3.70) and 4.33 (95% CI 1.47–12.77) times, respectively.

### 3.3. Clinical Manifestations of COVID-19

Table 2 presents the characteristics of COVID-19 symptoms according to vaccination status. Fever was less frequent among vaccinated women, with a significant difference between groups (46.6% vs. 58.9%, *p* = 0.005).

Women in the vaccinated group also presented lower odds of having any respiratory symptom compared to those in the unvaccinated group (OR 0.51, 95% CI 0.36–0.71). Likewise, COVID-19 vaccination was associated with a lower rate of dyspnea (OR 0.53; 95% CI 0.39–0.73; *p* < 0.001), respiratory discomfort (OR 0.62; 95% CI 0.46–0.86; *p* = 0.004) and desaturation (OR 0.53; 95% CI 0.39–0.73; *p* < 0.001). 

### 3.4. COVID-19 Adverse Outcomes

Table 3 presents the main COVID-19-related outcomes according to the vaccination status. Intensive care unit (ICU) admission rates were significantly lower among women in the vaccinated group than those in the unvaccinated group (23.5% vs. 37.4%; *p* < 0.001; OR 0.52; 95% CI 0.36–0.73). Although there was no difference regarding the length of time in the ICU between groups, there was a tendency for this to be shorter among vaccinated women (9.38 ± 9.30 and 13.09 ± 12.22 days; *p* = 0.090).

Rates of intubation were lower in the vaccinated group (4.8% vs. 18.8%; *p* < 0.001; OR 0.22; 95% CI. 11–0.43). The rates of maternal mortality were lower in vaccinated women (3.0% vs. 14.1%; *p* < 0.001; OR 0.188 (95% CI 0.083–0.428)) compared to unvaccinated women.

Lastly, PSM was conducted to account for confounding factors to verify whether the effect on outcome could be attributed to the COVID-19 vaccine (Table 3). The three main outcomes (ICU admission, intubation, and death) remained statistically less frequent in the vaccinated group than those in the unvaccinated group after balancing the covariates using this analysis.

NNTs for ICU admission, intubation, and death were 7, 7, and 9, respectively. Under the Brazilian health system, the daily ICU cost is BRL 1600 (USD 283.70), meaning a cost of over BRL 20,864 (USD 3699.30) for the average 13-day stay. In contrast, a 2-dose regiment of the vaccine costs BRL 116.40 (USD 20.82).

## 4. Discussion

In hospitalized pregnant and postpartum women with severe COVID-19, those who had received two doses of a COVID-19 vaccine had a 46% reduction in the odds of ICU admission, an 81% reduction in the odds of invasive ventilatory support, and an 80% reduction in the odds of death compared to those who did not receive any COVID-19 vaccination.

To the best of our knowledge, this is the first study to examine the association between COVID-19 vaccines and maternal mortality by SARS-CoV-2-induced SARS in women with severe disease. These results should encourage and reinforce the importance of COVID-19 vaccination in the obstetric population.

Despite these promising results, COVID-19 vaccination rates in pregnant and postpartum women remain low [6,32]. In Brazil, by 29 March 2022, 899,043 (40.3%) pregnant and postpartum women were fully vaccinated [6]. Reasons for this low uptake include maternal fear of exposing the fetus to vaccines, a desire to wait for more information regarding the safety and effectiveness of vaccines in pregnancy, and concerns related to the rapid development of the vaccines [10,33]. However, vaccine hesitancy is a nuanced phenomenon also affected by, for example, religious reasons, financial resources, and educational aspects [8,10,33].

Excluding pregnant and postpartum women (a high-risk group for respiratory infections) as a priority group in a vaccine RCT means restricting their opportunity to receive adequate care during a pandemic. Consequently, wrong decisions can occur due to a lack of robust evidence [8,10,11,32,33]. Pregnant and postpartum women should have the opportunity to enroll in RCTs, in agreement with the ethical principles of research, the characteristics of vaccines, and the context in which the RCT is conducted [10,12,33].

Observational studies and surveillance data concerning vaccination during pregnancy are reassuring. Considering that COVID-19 vaccines are novel, and the majority of clinical trials excluded pregnant and postpartum women, programs that monitor the safety, effectiveness, and side effects of COVID-19 vaccination, and adverse pregnancy outcomes, are extremely important [34,35]. This information could guide obstetricians to recommend vaccination to pregnant women and to improve its uptake, thereby overcoming vaccine hesitance [34]. In Brazil, it is compulsory that every adverse vaccine event is reported to the Ministry of Health [36].

Most of the subjects received the Pfizer/BioNTech or Moderna vaccines, and available data do not demonstrate adverse outcomes or side effects of vaccination during pregnancy [20,22,37,38,39,40,41]. Shimakaburo et al. evaluated the outcomes of 827 completed pregnancies. They reported that the frequencies of pregnancy loss, preterm birth, and small size for gestational age did not differ from those before the beginning of the pandemic [17]. However, none of these studies reported the protective effect of vaccines on the severity of clinical manifestations and mortality rate of women with severe COVID-19, as demonstrated in the present study.

One of the limitations of this study is that those who chose to be vaccinated may be more likely to have healthier behaviors, such as wearing a mask and maintaining social distance, which could confer additional protection in the vaccinated group. However, we enrolled women with established severe COVID-19 disease. In our study, women with more risk factors were also more likely to be vaccinated. Despite their otherwise higher risk status, they were less likely to require ICU admission or ventilation, or to die.

As demonstrated in the present study, there was a higher frequency of individuals with a lower educational level among the unvaccinated group, and those with easier access to health care programs may be more likely to avoid worse outcomes. Previous studies also reported some of these social features [32].

Regarding clinical manifestations of SARS-CoV-2 infection, our study found that the more severe symptoms (dyspnea, respiratory discomfort, and desaturation) were more prevalent among women in the unvaccinated group.

PSM analysis confirmed these findings in light of confounders. In addition, the NNT evaluation showed that only seven hospitalized pregnant women needed to be vaccinated to avoid one ICU admission. This finding could have a possible economic impact for the health system, especially for those living in low and middle-income countries.

This is an observational and retrospective study based on a standard case report form, and it depends on the adequacy and completeness of notification. We had to exclude all subjects without information regarding vaccination status (28.9%) and those without a second-dose date (11.7%). Only valid cases of the variables involved in analyses were considered. However, statistical analyses were also performed after the imputation of missing data; the results were similar, and the inference did not change (Supplemental Materials). Subjects for analyses were rigorously selected according to the availability of completed data, particularly confirmation of acute COVID-19 infection, the exclusion of H1N1 coinfection, and the date of the second dose of COVID-19 vaccination, thus ensuring that vaccination preceded clinical presentation.

The SIVEP-Gripe database only gathers information for severe acute respiratory infections. Hence, we do not know what the protective effect of vaccination is in pregnant individuals with mild symptoms.

## 5. Conclusions

The COVID-19 vaccine offers a protective effect against ICU admission, intubation, and death in hospitalized pregnant and postpartum women with severe SARS-CoV-2-induced SARS.

## Figures and Tables

**Table 1:** Baseline characteristics of subjects according to vaccination status.

Variables	Vaccinated (*n* = 200)	Unvaccinated (*n* = 2084)	*p*-Value
Age (years); mean ± sd ^$^	31.44 ± 7.72	29.72 ± 7.09	***0.003* ^1^**
Self-reported ethnicity, *n* (%) White Nonwhite			0.106 ^2^
	111/183 (60.7)	1022/1888 (54.1)	
	72/183 (39.3)	866/1888 (45.9)	
Comorbidities, *n* (%):			
Cardiac	22/93 (23.7)	104/842 (12.4)	***0.004* ^2^**
Diabetes mellitus	21/94 (22.3)	144/852 (16.9)	0.239 ^2^
Hematologic	2/89 (2.2)	7/814 (0.9)	0.219 ^3^
Obesity	14/90 (15.6)	191/871 (21.9)	0.204 ^2^
Asthma	9/90 (10.0)	65/832 (7.8)	0.602 ^2^
Hepatic	1/89 (1.1)	3/810 (0.4)	0.341 ^3^
Neurologic	2/88 (2.3)	9/811 (1.1)	0.293 ^3^
Other lung diseases	3/89 (3.4)	9/814 (1.1)	0.105 ^3^
Immunosuppression	5/89 (5.6)	11/812 (1.4)	***0.015* ^3^**
Renal	2/89 (2.2)	9/806 (1.1)	0.300 ^3^
Education, *n* (%)			0.116 ^2^
Up to 9 years	20/101 (19.8)	279/1044 (26.7)	
From 9 to 12 years	55/101 (54.5)	573/1044 (54.9)	
Over 12 years	26/101 (25.7)	192/1044 (18.4)	
Residence area, *n* (%)			0.850 ^3^
Urban	179/188 (95.2)	1838/1957 (93.9)	
Periurban	0 (0.0)	6/1957 (0.3)	
Rural	9/188 (4.8)	113/1957 (5.8)	

*n*: number; sd: standard deviation; ^1^: Student’s *t*-test; ^2^: chi-square test; ^3^: Fisher’s exact test; ^$^: Cohen’s d = −0.24 (95% CI −0.39–0.10—small).

**Table 2:** Characteristics of COVID-19 symptoms by vaccination status.

Variables	Vaccinated (*n* = 200)	Unvaccinated (*n* = 2084)	OR (95% CI)
Symptoms, *n* (%)			
Fever	82/176 (46.6)	1077/1860 (58.9)	***0.63* (*0.47*–*0.86*)**
Cough	137/182 (75.3)	1532/1938 (79.1)	0.81 (0.57–1.15)
Sore throat	33/159 (20.8)	447/1710 (26.1)	0.74 (0.50–1.10)
Dyspnea	97/173 (56.1)	1349/1914 (70.5)	***0.53* (*0.39*–*0.73*)**
Respiratory discomfort	77/172 (44.8)	1028/1820 (56.5)	***0.62* (*0.46*–*0.86*)**
Desaturation	69/172 (40.1)	1025/1840 (55.7)	***0.53* (*0.39*–*0.73*)**
Diarrhea	16/159 (10.1)	189/1677 (11.3)	0.88 (0.51–1.51)
Vomiting	16/159 (10.1)	202/1682 (12.0)	0.82 (0.48–1.40)
Abdominal pain	18/156 (11.5)	164/1658 (9.9)	1.19 (0.71–1.99)
Fatigue	53/163 (32.5)	590/1737 (34.0)	0.94 (0.67–1.32)
Loss of smell	33/160 (20.6)	310/1682 (18.4)	1.15 (0.77–1.72)
Loss of taste	30/159 (18.9)	287/1687 (16.8)	1.15 (0.76–1.74)
Any respiratory symptom	127/183 (69.4)	1618/1979 (81.8)	***0.51* (*0.36*–*0.71*)**
Any symptom	178/194 (91.8)	1980/2058 (96.2)	***0.44* (*0.25*–*0.77*)**

CI: confidence interval; *n*: number; OR: odds ratio.

**Table 3:** Characteristics of COVID-19 outcomes according to vaccination status, overall and after propensity score matching.

Variables *n* (%)	Vaccinated (*n* = 200)	Unvaccinated (*n* = 2084)	OR (95%CI)	Vaccinated (*n* = 200) PSM	Unvaccinated (*n* = 200) PSM	OR (95% CI) PSM
ICU admission	44/187 (23.5)	740/1979 (37.4)	***0.52* (*0.36*–*0.73*)**	44/187 (23.5)	69/190 (36.3)	***0.54* (*0.34*–*0.85*)**
Intubation	9/189 (4.8)	368/1959 (18.8)	***0.22* (*0.11*–*0.43*)**	9/189 (4.8)	39/189 (20.6)	***0.19* (*0.09*–*0.41*)**
Final outcome,						
Cure Death	194/200 (97.0) 6/200 (3.0)	1790/2084 (85.9) 294/2084 (14.1)	***0.188* (*0.083*–*0.428*)**	194/200 (97.0) 6/200 (3.0)	174/200 (87.0) 26/200 (13.0)	***0.207* (*0.0.83*–*0.515*)**

CI: confidence interval; *n*: number; OR: odds ratio; PSM: propensity score matching.

## Data Availability

Codes used to support the findings of this study and supplemental material are available in the GitHub repository at https://github.com/observatorioobstetrico/sup_mat_COVID-19-vaccines-confer-protection-in-hospitalized-pregnant-amd-postpartum-women-with-sever accessed on 5 April 2022. Datasets are available at https://www.kaggle.com/agatharodrigues/covid19-vaccine-maternal-population accessed on 5 April 2022.

## Supplementary Materials

The following supporting information can be downloaded at: https://www.mdpi.com/article/10.3390/vaccines10050749/s1, Text S1: Documentation of the article ‘COVID-19 vaccines confer protection in hospitalized pregnant and postpartum women with severe COVID-19’.
