# Supplementary material for: COVID-19 Vaccines Confer Protection in Hospitalized Pregnant and Postpartum Women with Severe COVID-19: A Retrospective Cohort Study
Source: Vaccines (Basel). 2022 May 10;10(5):749. doi: 10.3390/vaccines10050749 (PMC9146232; doi:10.3390/vaccines10050749)
Supplement: Supplementary file 1 [file vaccines-10-00749-s001.zip › vaccines-1692394-supplementary.pdf]

Text S1. Documentation of the article 'COVID-19 vaccines confer protection in hospitalized pregnant and postpartum women with severe COVID-19'

Codes and outputs

Feb 10, 2022

Sumário

Description **2**

About the database and R packages used **2**

Case selection and data treatment **4**

Propensity Scoring Method (PSM) - information about date of second dose **36**

Multivariate Imputation by Chained Equations (MICE) **43**

Description

This file presents the documentation of the analysis of article "COVID-19 vaccines confer protection in hospitalized pregnant and postpartum women with severe COVID-19".

About the database and R packages used

The data are analyzed using the free-software R (https://www.R-project.org) in version 4.0.3. Next, we present and load the libraries used in the data analysis process.

```r
#load packages
loadlibrary <- function(x) {
  if (!require(x, character.only = TRUE)) {
    install.packages(x, dependencies = T)
    if (!require(x, character.only = TRUE))
      stop("Package not found")
  }
}

packages <-
  c(
    "readr",
    "readxl",
    "janitor",
    "dplyr",
    "forcats",
    "stringr",
    "lubridate",
    "summarytools",
    "magrittr",
    "questionr",
    "knitr",
    "data.table",
    "modelsummary",
    "kableExtra",
    "DescTools",
    "effectsize",
    "WeightIt",
    "MatchIt",
    "ggplot2",
    "ggpubr",
    "naniar",
    "mice",
    "miceafter",
    "VIM",
    "miceadds"
  )

lapply(packages, loadlibrary)
```

One can see below the functions that will be used in the data analysis.

```r
#functions for summary measures
media <- function(x)
  mean(x, na.rm = TRUE)
mediana <- function(x)
  median(x, na.rm = TRUE)
DP <- function(x)
  sd(x, na.rm = TRUE)
minimo <- function(x)
  base::min(x, na.rm = TRUE)
maximo <- function(x)
  base::max(x, na.rm = TRUE)
q25 <- function(x)
  stats::quantile(x, p = 0.25, na.rm = TRUE)
q75 <- function(x)
  stats::quantile(x, p = 0.75, na.rm = TRUE)
IQR <- function(x)
  round(q75(x) - q25(x), 2)
n <- function(x)
  sum(!is.na(x))
```

This is a retrospective cohort study using the data from the Influenza Epidemiological Surveillance Information System, SIVEP-Gripe (Sistema de Informação de Vigilância Epidemiológica da Gripe) database.

The SIVEP-Gripe is a nationwide surveillance database created to monitor severe acute respiratory infections and data on virus circulation and respiratory infections in Brazil.

The period analyzed comprises epidemiological data from 2021, with a database obtained on December 2, 2021 on the website https://opendatasus.saude.gov.br. The dataset can be obtained at https://www.kaggle.com/agatharodrigues/covid19-vaccine-maternal-population. It is loaded below:

```r
#loading the datasets
#2021
dados <- read_delim(
  "INFLUD21-29-11-2021.csv",
  ";",
  escape_double = FALSE,
  locale = locale(encoding = "ISO-8859-2"),
  trim_ws = TRUE
)

#Create case year variable
dados <-  dados %>%
  dplyr::mutate(
    dt_sint = as.Date(DT_SIN_PRI, format = "%d/%m/%Y"), #date of first symptoms
    dt_nasc = as.Date(DT_NASC, format = "%d/%m/%Y"), #date of birth
    dt_vac_gripe = as.Date(DT_UT_DOSE, format = "%d/%m/%Y"), #date of Influenza vaccine
    ano = lubridate::year(dt_sint), #year of the case
  )
```

There are 1625471 observations in the database. To see the dictionary of variables, access (in Portuguese): https://opendatasus.saude.gov.br/dataset/ae90fa8f-3e94-467e-a33f-94adbb66edf8/resource/8f571374-c555-4ec0-8e44-00b1e8b11c25/download/dicionario-de-dados-srag-hospitalizado-27.07.2020-final.pdf

Case selection and data treatment

The first filter is to select cases from May 02, 2021 (18th epidemiological week of symptoms of 2021) to November 27, 2021 (epidemiological week 47 of 2021).

```
#selection of cases from 18th epidemiological week of symptoms (May 2, 2021)
#to November 27, 2021 (week 43 of 2021).
sem1 <- 18
sem2 <- 47

dados1 <- dados %>%
  filter(SEM_PRI >= sem1 &  SEM_PRI <= sem2)
```

There are 756681 observations in the database after selection of valid years.

The next selection is female:

```
#filtering F
dados2 <- filter(dados1, CS_SEXO == "F")
```

There are 338892 observations in the database.

Selection of women of childbearing age (10 to 55 years):

```
#creating the age variable as the difference between dt_sint and dt_nasc.
#In cases without dt_nasc, we consider
#the NU_AGE_N field
dados2 <- dados2 %>%
  mutate(
         idade = as.period(interval(start = dt_nasc, end = dt_sint))$year,
         age = ifelse(is.na(idade), NU_IDADE_N, idade)
  )

#Filtering of cases aged 55 and under
dados3 <- dados2 %>%
  filter(age > 9 & age <= 55)
```

There are 141540 observations in the database.

The next step is to identify pregnant and postpartum people (variable `classi_gesta_puerp`) and then select only those cases.

```
#Creating the classification variable if pregnant, postpartum and
##neither pregnant nor postpartum
dados3 <- dados3 %>%
  mutate(
    classi_gesta_puerp = case_when(
      CS_GESTANT == 1  ~ "1tri",
      CS_GESTANT == 2  ~ "2tri",
      CS_GESTANT == 3  ~ "3tri",
      CS_GESTANT == 4  ~ "IG_ig",
      CS_GESTANT == 5 &
        PUERPERA == 1 ~ "puerp",
      CS_GESTANT == 9 & PUERPERA == 1 ~ "puerp",
```

```
      TRUE ~ "no"
    )
  )

freq(dados3$classi_gesta_puerp)
```

```
##              n    % val%
## 1tri       800  0.6  0.6
## 2tri      2110  1.5  1.5
## 3tri      4958  3.5  3.5
## IG_ig      368  0.3  0.3
## no      131497 92.9 92.9
## puerp     1807  1.3  1.3
```

```
#filtering only pregnant and postpartum women
dados4 <- dados3 %>%
  filter(classi_gesta_puerp != "no")
```

There are 10043 observations in the database.

We selected only confirmed cases of COVID-19.

```
dados4 <- dados4 %>%
  mutate(
    classi_fin = case_when(
      CLASSI_FIN == 5 ~ "covid",
      TRUE ~ "no"
    )
  )


#filtering only covid cases
dados5 <- dados4 %>%
    filter(CLASSI_FIN == 5)
```

There are 5806 observations in the database.

Now let's select the cases of COVID by PCR or antigen, but which are also not positive for Influenza.

```
#COVID case diagnosed by PCR
dados5 <- dados5 %>%
  mutate(pcr_covid_SN = case_when(
    (PCR_SARS2 == 1) |
      (
        str_detect(DS_PCR_OUT, "SARS|COVID|COV|CORONA|CIVID")
      ) ~ "yes",
    TRUE ~ "no"
  ))

#Influenza case diagnosed by PCR
dados5 <- dados5 %>%
  mutate(pcr_influenza_SN = case_when(
    (POS_PCRFLU == 1) |
```

```
      (
        str_detect(DS_PCR_OUT, "INFLU|INFLUENZA")
      ) ~ "yes",
    TRUE ~ "no"
  ))

with(dados5, table(pcr_influenza_SN, pcr_covid_SN))
```

```
##                  pcr_covid_SN
## pcr_influenza_SN   no  yes
##              no  2806 2999
##              yes    1    0
```

There is no case that is positive for COVID and for Influenza by PCR.

```
#Case of COVID diagnosed by antigen
dados5 <- dados5 %>%
  mutate(antigenio_covid_SN = case_when(
    (AN_SARS2 == 1) |
      (
        str_detect(DS_AN_OUT, "SARS|COVID|COV|CORONA|CIVID")
      ) ~ "yes",
    TRUE ~ "no"
  ))


#Influenza case diagnosed by antigen
dados5 <- dados5 %>%
  mutate(antigenio_influenza_SN = case_when(
    (POS_AN_FLU == 1) |
      (
        str_detect(DS_AN_OUT, "INFLU|INFLUENZA")
      ) ~ "yes",
    TRUE ~ "no"
  ))

with(dados5, table(antigenio_influenza_SN, antigenio_covid_SN))
```

```
##                        antigenio_covid_SN
## antigenio_influenza_SN   no  yes
##                    no  4306 1499
##                    yes    0    1
```

There is one positive case for COVID and for Influenza by antigen.

We will now select the cases of COVID confirmed by PCR or antigen.

```
with(dados5, table(pcr_covid_SN, antigenio_covid_SN))
```

```
##             antigenio_covid_SN
## pcr_covid_SN   no  yes
##          no  1518 1289
##          yes 2788  211
```

```r
#filtering only covid cases by PCR or antigen
dados6 <- dados5 %>%
    filter(pcr_covid_SN == "yes" | antigenio_covid_SN == "yes")
```

There are 4288 observations in the database.

Now it's time to remove cases that are also positive for Influenza.

```r
with(dados6, table(pcr_influenza_SN, antigenio_influenza_SN))
```

```
##                   antigenio_influenza_SN
## pcr_influenza_SN   no  yes
##              no  4287    0
##              yes    0    1
```

```r
#filtering only negative cases of Influenza by PCR or antigen
dados7 <- dados6 %>%
    filter(pcr_influenza_SN != "yes" & antigenio_influenza_SN != "yes")
```

There are 4287 observations in the database.

We will only select the finalized cases (death or cure). The variable that indicates the outcome is `EVOLUCAO`, with the categories: 1-Cure; 2-Death; 3- Death from other causes; 9-Ignored.

```r
with(dados7, freq(EVOLUCAO))
```

```
##        n    % val%
## 1  3351 78.2 84.9
## 2   487 11.4 12.3
## 3     8  0.2  0.2
## 9   100  2.3  2.5
## NA  341  8.0   NA
```

Let's select only the finalized cases:

```r
#filtering only completed cases
dados8 <- dados7 %>%
  filter((EVOLUCAO == 1 | EVOLUCAO == 2 | EVOLUCAO == 3) & !is.na(EVOLUCAO))


#creating the evolution variable
dados8 <- dados8 %>%
  mutate(death = case_when(
    EVOLUCAO == 1 ~ "cure",
    EVOLUCAO == 2 ~ "death",
    EVOLUCAO == 3 ~ "death"
  ))

with(dados8, freq(death))
```

```
##           n    % val%
## cure   3351 87.1 87.1
## death   495 12.9 12.9
```

There are 3846 observations in the database.

The variable that indicates whether the person received a vaccine against COVID-19 is `VACINA_COV`, with categories: 1-yes; 2-no; 9-ignored.

```
#Frequency table for VACINA_COV
with(dados8, freq(VACINA_COV))
```

```
##       n    % val%
## 1   650 16.9 18.7
## 2  2084 54.2 60.0
## 9   738 19.2 21.3
## NA  374  9.7   NA
```

Let's now group "NA" and "9" in the same category (NA - missing data) and label the valid categories.

```
#recoding the vaccine_cov variable
dados8 <- dados8 %>%
  mutate(vaccine_cov = case_when(
    VACINA_COV == 1 ~ "yes",
    VACINA_COV == 2 ~ "no",
    TRUE ~ NA_character_
  ))
#frequency table for vaccine_cov
with(dados8, freq(vaccine_cov))
```

```
##        n    % val%
## no  2084 54.2 76.2
## yes  650 16.9 23.8
## NA  1112 28.9   NA
```

The next step is filtering cases that we have information about COVID-19 vaccination. These data are analyzed in the following.

```
#Filtering cases with information about vaccination
data_final <- dados8 %>%
  filter(!is.na(vaccine_cov))
```

```
with(data_final, freq(vaccine_cov))
```

```
##        n    % val%
## no  2084 76.2 76.2
## yes  650 23.8 23.8
```

The variable `vaccine_cov` only indicates if the pregnant or postpartum women took the vaccine, regardless of the dose. There is no information on whether the person only took the first dose or the second. The closest we come to this is to consider the column `DOSE_2_COV`, which indicates the date of the second dose.

```
#Create second dose date variable
data_final <-  data_final %>%
  dplyr::mutate(
```

```
    dt_dose2_cov = as.Date(DOSE_2_COV, format = "%d/%m/%Y")
  )

#Create variable that indicates that it has the date of the second dose
data_final <-  data_final%>%
  dplyr::mutate(
    indic_dt_dose2_cov = ifelse(is.na(dt_dose2_cov) & !is.na(vaccine_cov), 0, ifelse(is.na(vaccine_cov)
  )

# first dose date frequency table
with(data_final, freq(indic_dt_dose2_cov, total =  TRUE))
```

```
##           n     %  val%
## 0      2534  92.7  92.7
## 1       200   7.3   7.3
## Total 2734 100.0 100.0
```

There is only information on the date of the second dose for 200 cases of 650 cases indicated as "yes" for COVID-19 vaccine.

Now we will analyze the not vaccinated group versus two dose vaccinated group.

```
#Create second dose date variable
data_final <- data_final %>%
  filter(vaccine_cov == "no" | (vaccine_cov == "yes" & indic_dt_dose2_cov == 1))

with(data_final, freq(vaccine_cov))
```

```
##         n    % val%
## no   2084 91.2 91.2
## yes   200  8.8  8.8
```

#Analysis

Epidemiologic characteristics

```
# Ethnicity
data_final <-  data_final %>%
  mutate(
    ethnicity = case_when(
      CS_RACA == 1 ~ "white",
      CS_RACA == 2 ~ "black",
      CS_RACA == 3 ~ "yellow",
      CS_RACA == 4 ~ "brown",
      CS_RACA == 5 ~ "indigenous",
      TRUE ~ NA_character_
    ),
    white_color = case_when(
      ethnicity == "white" ~ "yes",
      is.na(ethnicity) ~ NA_character_,
```

```r
      TRUE ~ "no"
    )
  )

# Education
data_final <-  data_final %>%
  mutate(
    education2 = case_when(
      CS_ESCOL_N <= 2 ~ "up to 9 years",
      CS_ESCOL_N == 3 ~ "from 9 to 12 years",
      CS_ESCOL_N == 4 ~ "over 12 years",
      TRUE ~ NA_character_
    )
  )
data_final$education2 <-
  factor(data_final$education2, levels = c("up to 9 years", "from 9 to 12 years", "over 12 years"))

# residence area
data_final <-  data_final %>%
  mutate(
    residence = case_when(
      CS_ZONA == 1 ~ "urban",
      CS_ZONA == 2 ~ "rural",
      CS_ZONA == 3 ~ "periurban",
      TRUE ~ NA_character_
    )
  )

# residence area 2 (grouping the categories urban and periurban)
data_final <-  data_final %>%
  mutate(
    residence2 = case_when(
      CS_ZONA == 1 ~ "urban/periurban",
      CS_ZONA == 2 ~ "rural",
      CS_ZONA == 3 ~ "urban/periurban",
      TRUE ~ NA_character_
    )
  )
data_final$residence2 <-
  factor(data_final$residence2, levels = c("rural", "urban/periurban"))
```

Ethnicity

```r
with(data_final, ctable(ethnicity, vaccine_cov,  prop = "c", useNA = "no", chisq = FALSE, OR = FALSE))
```

```
## Cross-Tabulation, Column Proportions
## ethnicity * vaccine_cov
## Data Frame: data_final
##
## ------------ ------------- --------------- -------------- ---------------
##                vaccine_cov              no            yes           Total
```

```
##    ethnicity
##        black                       96 (  5.1%)     6 (  3.3%)    102 (  4.9%)
##        brown                      753 ( 39.9%)    61 ( 33.3%)    814 ( 39.3%)
##   indigenous                        5 (  0.3%)     4 (  2.2%)      9 (  0.4%)
##        white                     1022 ( 54.1%)   111 ( 60.7%)   1133 ( 54.7%)
##       yellow                       12 (  0.6%)     1 (  0.5%)     13 (  0.6%)
##        Total                     1888 (100.0%)   183 (100.0%)   2071 (100.0%)
## ------------ ------------- --------------- -------------- ---------------
```

```
fisher.test(data_final$ethnicity, data_final$vaccine_cov)
```

```
##
##  Fisher's Exact Test for Count Data
##
## data:  data_final$ethnicity and data_final$vaccine_cov
## p-value = 0.007548
## alternative hypothesis: two.sided
```

White color

```
with(data_final, ctable(white_color, vaccine_cov,  prop = "c", useNA = "no", chisq = TRUE, OR = TRUE))
```

```
## Cross-Tabulation, Column Proportions
## white_color * vaccine_cov
## Data Frame: data_final
##
##
## ------------- ------------- --------------- -------------- ---------------
##                 vaccine_cov              no            yes           Total
##   white_color
##            no                  866 ( 45.9%)    72 ( 39.3%)    938 ( 45.3%)
##           yes                 1022 ( 54.1%)   111 ( 60.7%)   1133 ( 54.7%)
##         Total                 1888 (100.0%)   183 (100.0%)   2071 (100.0%)
## ------------- ------------- --------------- -------------- ---------------
##
## ----------------------------
##  Chi.squared   df   p.value
## ------------- ---- ---------
##    2.6088      1    0.1063
## ----------------------------
##
## ----------------------------------
##  Odds Ratio   Lo - 95%   Hi - 95%
## ------------ ---------- ----------
##     1.31        0.96       1.78
## ----------------------------------
```

Education (years)

```r
with(data_final, ctable(education2, vaccine_cov, prop = "c", useNA = "no", chisq = TRUE))
```

```
## Cross-Tabulation, Column Proportions
## education2 * vaccine_cov
## Data Frame: data_final
##
##
## -------------------- ------------- --------------- -------------- ---------------
##                        vaccine_cov              no            yes           Total
##           education2
##         up to 9 years                  279 ( 26.7%)    20 ( 19.8%)    299 ( 26.1%)
##    from 9 to 12 years                  573 ( 54.9%)    55 ( 54.5%)    628 ( 54.8%)
##        over 12 years                   192 ( 18.4%)    26 ( 25.7%)    218 ( 19.0%)
##                Total                  1044 (100.0%)   101 (100.0%)   1145 (100.0%)
## -------------------- ------------- --------------- -------------- ---------------
##
## ----------------------------
##  Chi.squared   df   p.value
## ------------- ---- ---------
##    4.3072      2    0.1161
## ----------------------------
```

Age

```r
datasummary((vaccine_cov) ~ age*(n+media+DP+mediana+q25+q75+IQR),
            data = data_final, output = 'markdown')
```

	n	media	DP	mediana	q25	q75	IQR
no	2084.00	29.72	7.09	30.00	25.00	35.00	10.00
yes	200.00	31.44	7.72	31.00	25.00	37.00	12.00

```r
#t-test
t.test(age ~ vaccine_cov, data = data_final)
```

```
##
##  Welch Two Sample t-test
##
## data:  age by vaccine_cov
## t = -3.0298, df = 232.39, p-value = 0.002724
## alternative hypothesis: true difference in means between group no and group yes is not equal to 0
## 95 percent confidence interval:
##  -2.8372657 -0.6012756
## sample estimates:
##  mean in group no mean in group yes
##          29.72073          31.44000
```

```r
#effect size
c_cohen <- cohens_d(age ~ as.factor(vaccine_cov), data=data_final)
c_cohen
```

```
## Cohen's d |         95% CI
## --------------------------
## -0.24     | [-0.39, -0.10]
##
## - Estimated using pooled SD.
```

```r
interpret_d(c_cohen$Cohens_d,rules="cohen1988")
```

```
## [1] "small"
## (Rules: cohen1988)
```

Residence area

```r
with(data_final, ctable(residence, vaccine_cov, prop = "c", useNA = "no", chisq = FALSE))
```

```
## Cross-Tabulation, Column Proportions
## residence * vaccine_cov
## Data Frame: data_final
##
## ----------- ------------- --------------- -------------- ---------------
##               vaccine_cov              no            yes           Total
##   residence
##   periurban                   6 (  0.3%)     0 (  0.0%)      6 (  0.3%)
##       rural                 113 (  5.8%)     9 (  4.8%)    122 (  5.7%)
##       urban                1838 ( 93.9%)   179 ( 95.2%)   2017 ( 94.0%)
##       Total                1957 (100.0%)   188 (100.0%)   2145 (100.0%)
## ----------- ------------- --------------- -------------- ---------------
```

```r
fisher.test(data_final$residence, data_final$vaccine_cov)
```

```
##
##  Fisher's Exact Test for Count Data
##
## data:  data_final$residence and data_final$vaccine_cov
## p-value = 0.8508
## alternative hypothesis: two.sided
```

Residence area 2 (grouping the categories urban and periurban)

```r
with(data_final, ctable(residence2, vaccine_cov, prop = "c", useNA = "no", chisq = TRUE, OR = TRUE))
```

```
## Cross-Tabulation, Column Proportions
## residence2 * vaccine_cov
```

```
## Data Frame: data_final
##
##
## ----------------- ------------- --------------- -------------- ---------------
##                     vaccine_cov              no            yes           Total
##        residence2
##             rural                  113 (  5.8%)     9 (  4.8%)    122 (  5.7%)
##   urban/periurban                 1844 ( 94.2%)   179 ( 95.2%)   2023 ( 94.3%)
##             Total                 1957 (100.0%)   188 (100.0%)   2145 (100.0%)
## ----------------- ------------- --------------- -------------- ---------------
##
## ----------------------------
##  Chi.squared   df   p.value
## ------------- ---- ---------
##    0.1546      1    0.6941
## ----------------------------
##
## ----------------------------------
##  Odds Ratio   Lo - 95%   Hi - 95%
## ------------ ---------- ----------
##     1.22        0.61       2.44
## ----------------------------------
```

Comorbities

```
#Cardiac
data_final <-  data_final %>%
  mutate(cardiac = case_when(CARDIOPATI == 1 ~ "yes",
                             CARDIOPATI == 2 ~ "no",
                             TRUE ~ NA_character_))

#Hematologic
data_final <-  data_final %>%
  mutate(hematologic = case_when(HEMATOLOGI == 1 ~ "yes",
                                 HEMATOLOGI == 2 ~ "no",
                                 TRUE ~ NA_character_))

#Hepatic
data_final <-  data_final %>%
  mutate(hepatic = case_when(HEPATICA == 1 ~ "yes",
                             HEPATICA == 2 ~ "no",
                             TRUE ~ NA_character_))

#Asthma
data_final <-  data_final %>%
  mutate(asthma = case_when(ASMA == 1 ~ "yes",
                            ASMA == 2 ~ "no",
                            TRUE ~ NA_character_))

#Diabetes
data_final <-  data_final %>%
  mutate(diabetes = case_when(DIABETES == 1 ~ "yes",
```

```
                              DIABETES == 2 ~ "no",
                              TRUE ~ NA_character_))

#Neurologic
data_final <-  data_final %>%
  mutate(neurologic = case_when(NEUROLOGIC == 1 ~ "yes",
                                NEUROLOGIC == 2 ~ "no",
                                TRUE ~ NA_character_))

#Pneumologic
data_final <-  data_final %>%
  mutate(pneumologic = case_when(PNEUMOPATI == 1 ~ "yes",
                                 PNEUMOPATI == 2 ~ "no",
                                 TRUE ~ NA_character_))

#Imunossupression
data_final <-  data_final %>%
  mutate(imuno = case_when(IMUNODEPRE == 1 ~ "yes",
                           IMUNODEPRE == 2 ~ "no",
                           TRUE ~ NA_character_))

#Renal
data_final <-  data_final %>%
  mutate(renal = case_when(RENAL == 1 ~ "yes",
                           RENAL == 2 ~ "no",
                           TRUE ~ NA_character_))

#Obesity
data_final <-  data_final %>%
  mutate(obesity = case_when(OBESIDADE == 1 ~ "yes",
                             OBESIDADE == 2 ~ "no",
                             TRUE ~ NA_character_))
```

Cardiac

```
with(data_final, ctable(vaccine_cov, cardiac, prop = "r", useNA = "no", chisq = TRUE, OR = TRUE))
```

```
## Cross-Tabulation, Row Proportions
## vaccine_cov * cardiac
## Data Frame: data_final
##
##
## ------------- --------- ------------- ------------- --------------
##                 cardiac            no           yes          Total
##   vaccine_cov
##            no               738 (87.6%)   104 (12.4%)   842 (100.0%)
##           yes                71 (76.3%)    22 (23.7%)    93 (100.0%)
##         Total               809 (86.5%)   126 (13.5%)   935 (100.0%)
## ------------- --------- ------------- ------------- --------------
##
## ----------------------------
```

```
##  Chi.squared   df   p.value
## ------------- ---- ---------
##    8.2348      1    0.0041
## ----------------------------
##
## ----------------------------------
##  Odds Ratio   Lo - 95%   Hi - 95%
## ------------ ---------- ----------
##     2.20        1.31       3.70
## ----------------------------------
```

Hematologic

```
with(data_final, ctable(vaccine_cov, hematologic, prop = "r", useNA = "no", chisq = FALSE, OR = TRUE))
```

```
## Cross-Tabulation, Row Proportions
## vaccine_cov * hematologic
## Data Frame: data_final
##
##
## ------------- ------------- ------------- ---------- --------------
##                 hematologic            no        yes          Total
##   vaccine_cov
##            no                 807 (99.1%)   7 (0.9%)   814 (100.0%)
##           yes                  87 (97.8%)   2 (2.2%)    89 (100.0%)
##         Total                 894 (99.0%)   9 (1.0%)   903 (100.0%)
## ------------- ------------- ------------- ---------- --------------
##
## ----------------------------------
##  Odds Ratio   Lo - 95%   Hi - 95%
## ------------ ---------- ----------
##     2.65        0.54      12.96
## ----------------------------------
```

```
fisher.test(data_final$hematologic, data_final$vaccine_cov)
```

```
##
##  Fisher's Exact Test for Count Data
##
## data:  data_final$hematologic and data_final$vaccine_cov
## p-value = 0.2198
## alternative hypothesis: true odds ratio is not equal to 1
## 95 percent confidence interval:
##   0.2642886 14.1850125
## sample estimates:
## odds ratio
##   2.646116
```

Diabetes

```
ctable(data_final$vaccine_cov, data_final$diabetes, chisq=TRUE, prop="r",  useNA = "no", OR = TRUE)
```

```
## Cross-Tabulation, Row Proportions
## vaccine_cov * diabetes
## Data Frame: data_final
##
##
## ------------- ---------- ------------- ------------- --------------
##                 diabetes            no           yes          Total
##   vaccine_cov
##            no              708 (83.1%)   144 (16.9%)   852 (100.0%)
##           yes               73 (77.7%)    21 (22.3%)    94 (100.0%)
##         Total              781 (82.6%)   165 (17.4%)   946 (100.0%)
## ------------- ---------- ------------- ------------- --------------
##
## ----------------------------
##  Chi.squared   df   p.value
## ------------- ---- ---------
##     1.382      1    0.2398
## ----------------------------
##
## ----------------------------------
##  Odds Ratio   Lo - 95%   Hi - 95%
## ------------ ---------- ----------
##     1.41        0.84       2.37
## ----------------------------------
```

Obesity

```
ctable(data_final$vaccine_cov, data_final$obesity, chisq=TRUE, prop="r",  useNA = "no", OR = TRUE)
```

```
## Cross-Tabulation, Row Proportions
## vaccine_cov * obesity
## Data Frame: data_final
##
##
## ------------- --------- ------------- ------------- --------------
##                 obesity            no           yes          Total
##   vaccine_cov
##            no             680 (78.1%)   191 (21.9%)   871 (100.0%)
##           yes              76 (84.4%)    14 (15.6%)    90 (100.0%)
##         Total             756 (78.7%)   205 (21.3%)   961 (100.0%)
## ------------- --------- ------------- ------------- --------------
##
## ----------------------------
##  Chi.squared   df   p.value
## ------------- ---- ---------
##    1.6129      1    0.2041
```

```
## ----------------------------
##
## ----------------------------------
##  Odds Ratio   Lo - 95%   Hi - 95%
## ------------ ---------- ----------
##     0.66        0.36       1.19
## ----------------------------------
```

Asthma

```r
ctable(data_final$vaccine_cov, data_final$asthma, chisq=TRUE, prop="r",  useNA = "no", OR = TRUE)
```

```
## Cross-Tabulation, Row Proportions
## vaccine_cov * asthma
## Data Frame: data_final
##
##
## ------------- -------- ------------- ------------ --------------
##                 asthma            no          yes          Total
##   vaccine_cov
##            no            767 (92.2%)   65 ( 7.8%)   832 (100.0%)
##           yes             81 (90.0%)    9 (10.0%)    90 (100.0%)
##         Total            848 (92.0%)   74 ( 8.0%)   922 (100.0%)
## ------------- -------- ------------- ------------ --------------
##
## ----------------------------
##  Chi.squared   df   p.value
## ------------- ---- ---------
##    0.2718      1    0.6021
## ----------------------------
##
## ----------------------------------
##  Odds Ratio   Lo - 95%   Hi - 95%
## ------------ ---------- ----------
##     1.31        0.63       2.73
## ----------------------------------
```

Hepatic

```r
ctable(data_final$vaccine_cov, data_final$hepatic, chisq=FALSE, prop="r",  useNA = "no", OR = TRUE)
```

```
## Cross-Tabulation, Row Proportions
## vaccine_cov * hepatic
## Data Frame: data_final
##
##
## ------------- --------- -------------- ---------- --------------
##                 hepatic             no        yes          Total
##   vaccine_cov
```

```
##            no             807 ( 99.6%)   3 (0.4%)   810 (100.0%)
##           yes              88 ( 98.9%)   1 (1.1%)    89 (100.0%)
##         Total             895 ( 99.6%)   4 (0.4%)   899 (100.0%)
## ------------- ---------- -------------- ---------- --------------
##
## ----------------------------------
##  Odds Ratio   Lo - 95%   Hi - 95%
## ------------ ---------- ----------
##     3.06        0.31      29.70
## ----------------------------------
```

```
fisher.test(data_final$vaccine_cov, data_final$hepatic)
```

```
##
##  Fisher's Exact Test for Count Data
##
## data:  data_final$vaccine_cov and data_final$hepatic
## p-value = 0.3415
## alternative hypothesis: true odds ratio is not equal to 1
## 95 percent confidence interval:
##   0.05758056 38.46642499
## sample estimates:
## odds ratio
##   3.050859
```

Neurologic

```
ctable(data_final$vaccine_cov, data_final$neurologic, chisq=FALSE, prop="r",  useNA = "no", OR = TRUE)
```

```
## Cross-Tabulation, Row Proportions
## vaccine_cov * neurologic
## Data Frame: data_final
##
##
## ------------- ------------ ------------- ----------- --------------
##                 neurologic            no         yes          Total
##   vaccine_cov
##            no                802 (98.9%)    9 (1.1%)   811 (100.0%)
##           yes                 86 (97.7%)    2 (2.3%)    88 (100.0%)
##         Total                888 (98.8%)   11 (1.2%)   899 (100.0%)
## ------------- ------------ ------------- ----------- --------------
##
## ----------------------------------
##  Odds Ratio   Lo - 95%   Hi - 95%
## ------------ ---------- ----------
##     2.07        0.44       9.75
## ----------------------------------
```

```
fisher.test(data_final$vaccine_cov, data_final$neurologic)
```

```
##
##  Fisher's Exact Test for Count Data
##
## data:  data_final$vaccine_cov and data_final$neurologic
## p-value = 0.2936
## alternative hypothesis: true odds ratio is not equal to 1
## 95 percent confidence interval:
##   0.2143274 10.2318114
## sample estimates:
## odds ratio
##   2.070139
```

Pneumologic

```
ctable(data_final$vaccine_cov, data_final$pneumologic, chisq=FALSE, prop="r",  useNA = "no", OR = TRUE)
```

```
## Cross-Tabulation, Row Proportions
## vaccine_cov * pneumologic
## Data Frame: data_final
##
##
## ------------- ------------- ------------- ----------- --------------
##                 pneumologic            no         yes          Total
##   vaccine_cov
##            no                 805 (98.9%)    9 (1.1%)   814 (100.0%)
##           yes                  86 (96.6%)    3 (3.4%)    89 (100.0%)
##         Total                 891 (98.7%)   12 (1.3%)   903 (100.0%)
## ------------- ------------- ------------- ----------- --------------
##
## ----------------------------------
##  Odds Ratio   Lo - 95%   Hi - 95%
## ------------ ---------- ----------
##     3.12        0.83      11.74
## ----------------------------------
```

```
fisher.test(data_final$vaccine_cov, data_final$pneumologic)
```

```
##
##  Fisher's Exact Test for Count Data
##
## data:  data_final$vaccine_cov and data_final$pneumologic
## p-value = 0.1059
## alternative hypothesis: true odds ratio is not equal to 1
## 95 percent confidence interval:
##   0.5323484 12.7897697
## sample estimates:
## odds ratio
##   3.114462
```

Imunossupression

```r
ctable(data_final$vaccine_cov, data_final$imuno, chisq=FALSE, prop="r", useNA = "no", OR = TRUE)
```

```
## Cross-Tabulation, Row Proportions
## vaccine_cov * imuno
## Data Frame: data_final
##
##
## ------------- ------- ------------- ----------- --------------
##                 imuno            no         yes          Total
##   vaccine_cov
##            no           801 (98.6%)   11 (1.4%)   812 (100.0%)
##           yes            84 (94.4%)    5 (5.6%)    89 (100.0%)
##         Total           885 (98.2%)   16 (1.8%)   901 (100.0%)
## ------------- ------- ------------- ----------- --------------
##
## ----------------------------------
##  Odds Ratio   Lo - 95%   Hi - 95%
## ------------ ---------- ----------
##     4.33        1.47      12.77
## ----------------------------------
```

```r
fisher.test(data_final$imuno, data_final$vaccine_cov)
```

```
##
##  Fisher's Exact Test for Count Data
##
## data:  data_final$imuno and data_final$vaccine_cov
## p-value = 0.01526
## alternative hypothesis: true odds ratio is not equal to 1
## 95 percent confidence interval:
##   1.149292 13.896222
## sample estimates:
## odds ratio
##    4.32279
```

Renal

```r
ctable(data_final$vaccine_cov, data_final$renal, chisq=FALSE, prop="r",  useNA = "no", OR = TRUE)
```

```
## Cross-Tabulation, Row Proportions
## vaccine_cov * renal
## Data Frame: data_final
##
##
## ------------- ------- ------------- ----------- --------------
##                 renal            no         yes          Total
##   vaccine_cov
```

```
##            no           797 (98.9%)    9 (1.1%)   806 (100.0%)
##           yes            87 (97.8%)    2 (2.2%)    89 (100.0%)
##         Total           884 (98.8%)   11 (1.2%)   895 (100.0%)
## ------------- ------- ------------- ----------- --------------
##
## ----------------------------------
##  Odds Ratio   Lo - 95%   Hi - 95%
## ------------ ---------- ----------
##     2.04        0.43       9.57
## ----------------------------------
```

```
fisher.test(data_final$renal, data_final$vaccine_cov)
```

```
##
##  Fisher's Exact Test for Count Data
##
## data:  data_final$renal and data_final$vaccine_cov
## p-value = 0.3002
## alternative hypothesis: true odds ratio is not equal to 1
## 95 percent confidence interval:
##   0.21058 10.04877
## sample estimates:
## odds ratio
##   2.033657
```

Symptoms

```
# Fever
data_final <-  data_final %>%
  mutate(fever = case_when(FEBRE == 1 ~ "yes",
                           FEBRE == 2 ~ "no",
                           TRUE ~ NA_character_))

# Cough
data_final <-  data_final %>%
  mutate(cough = case_when(TOSSE == 1 ~ "yes",
                           TOSSE == 2 ~ "no",
                           TRUE ~ NA_character_))

# Sore throat
data_final <-  data_final %>%
  mutate(sore_throat = case_when(GARGANTA == 1 ~ "yes",
                                 GARGANTA == 2 ~ "no",
                                 TRUE ~ NA_character_))

# Dyspnea
data_final <-  data_final %>%
  mutate(dyspnea = case_when(DISPNEIA == 1 ~ "yes",
                             DISPNEIA == 2 ~ "no",
                             TRUE ~ NA_character_))
```

```r
# Respiratory discomfort
data_final <-  data_final %>%
  mutate(resp_disc = case_when(DESC_RESP == 1 ~ "yes",
                               DESC_RESP == 2 ~ "no",
                               TRUE ~ NA_character_))

# Desaturation
data_final <-  data_final %>%
  mutate(desaturation = case_when(SATURACAO == 1 ~ "yes",
                                  SATURACAO == 2 ~ "no",
                                  TRUE ~ NA_character_))

# Diarrhea
data_final <-  data_final %>%
  mutate(diarrhea = case_when(DIARREIA == 1 ~ "yes",
                              DIARREIA == 2 ~ "no",
                              TRUE ~ NA_character_))

# Vomit
data_final <-  data_final %>%
  mutate(vomit = case_when(VOMITO == 1 ~ "yes",
                           VOMITO == 2 ~ "no",
                           TRUE ~ NA_character_))

# Abdominal pain
data_final <-  data_final %>%
  mutate(abd_pain = case_when(DOR_ABD == 1 ~ "yes",
                              DOR_ABD == 2 ~ "no",
                              TRUE ~ NA_character_))

# Fatigue
data_final <-  data_final %>%
  mutate(fatigue = case_when(FADIGA == 1 ~ "yes",
                             FADIGA == 2 ~ "no",
                             TRUE ~ NA_character_))

# Olfactory loss
data_final <-  data_final %>%
  mutate(olfac_loss = case_when(PERD_OLFT == 1 ~ "yes",
                                PERD_OLFT == 2 ~ "no",
                                TRUE ~ NA_character_))

# Loss of taste
data_final <-  data_final %>%
  mutate(loss_taste = case_when(PERD_PALA == 1 ~ "yes",
                                PERD_PALA == 2 ~ "no",
                                TRUE ~ NA_character_))

# Any respiratory symptom
df <- data_final %>%
  select(dyspnea,fatigue,desaturation,resp_disc)

soma <- function(x){
```

```
  if (sum(is.na(x))==4)
    return(NA_character_)
  else
    return(sum(!is.na(x) & x=="yes"))
}
data_final$qt_sintomas_resp_aux <- apply(df,1,soma)

data_final <-  data_final %>%
  mutate(resp_symp = case_when(qt_sintomas_resp_aux >=1 ~ "yes",
                               qt_sintomas_resp_aux ==0 ~ "no",
                               TRUE ~ NA_character_))
# Any symptom
df <- data_final %>%
  select(dyspnea,fatigue,desaturation,resp_disc,
         fever,cough,sore_throat,diarrhea,vomit,abd_pain,olfac_loss,loss_taste)
soma <- function(x){
  if (sum(is.na(x))==12)
    return(NA_character_)
  else
    return(sum(!is.na(x) & x=="yes"))
}
data_final$qt_sintomas_aux <- apply(df,1,soma)

data_final <-  data_final %>%
  mutate(symptom = case_when(qt_sintomas_aux >= 1 ~ "yes",
                             qt_sintomas_aux == 0 ~ "no",
                             TRUE ~ NA_character_))
```

Fever

```
with(data_final, ctable(vaccine_cov, fever, prop = "r", useNA = "no", chisq = TRUE, OR=TRUE))
```

```
## Cross-Tabulation, Row Proportions
## vaccine_cov * fever
## Data Frame: data_final
##
##
## ------------- ------- ------------- -------------- ---------------
##                 fever            no            yes           Total
##   vaccine_cov
##            no            783 (42.1%)   1077 (57.9%)   1860 (100.0%)
##           yes             94 (53.4%)     82 (46.6%)    176 (100.0%)
##         Total            877 (43.1%)   1159 (56.9%)   2036 (100.0%)
## ------------- ------- ------------- -------------- ---------------
##
## ----------------------------
##  Chi.squared   df   p.value
## ------------- ---- ---------
##    7.9362      1    0.0048
## ----------------------------
##
```

```
## ----------------------------------
##  Odds Ratio   Lo - 95%   Hi - 95%
## ------------ ---------- ----------
##     0.63        0.47       0.86
## ----------------------------------
```

Cough

```r
with(data_final, ctable(vaccine_cov, cough, prop = "r", useNA = "no", chisq = TRUE, OR=TRUE))
```

```
## Cross-Tabulation, Row Proportions
## vaccine_cov * cough
## Data Frame: data_final
##
##
## ------------- ------- ------------- -------------- ---------------
##                 cough            no            yes           Total
##   vaccine_cov
##            no            406 (20.9%)   1532 (79.1%)   1938 (100.0%)
##           yes             45 (24.7%)    137 (75.3%)    182 (100.0%)
##         Total            451 (21.3%)   1669 (78.7%)   2120 (100.0%)
## ------------- ------- ------------- -------------- ---------------
##
## ----------------------------
##  Chi.squared   df   p.value
## ------------- ---- ---------
##    1.1998      1    0.2734
## ----------------------------
##
## ----------------------------------
##  Odds Ratio   Lo - 95%   Hi - 95%
## ------------ ---------- ----------
##     0.81        0.57       1.15
## ----------------------------------
```

Sore throat

```r
with(data_final, ctable(vaccine_cov, sore_throat, prop = "r", useNA = "no", chisq = TRUE, OR=TRUE))
```

```
## Cross-Tabulation, Row Proportions
## vaccine_cov * sore_throat
## Data Frame: data_final
##
##
## ------------- ------------- -------------- ------------- ---------------
##                 sore_throat             no           yes           Total
##   vaccine_cov
##            no                 1263 (73.9%)   447 (26.1%)   1710 (100.0%)
##           yes                  126 (79.2%)    33 (20.8%)    159 (100.0%)
```

```
##         Total                  1389 (74.3%)   480 (25.7%)   1869 (100.0%)
## ------------- ------------- -------------- ------------- ---------------
##
## ----------------------------
##  Chi.squared   df   p.value
## ------------- ---- ---------
##    1.9375      1    0.1639
## ----------------------------
##
## ----------------------------------
##  Odds Ratio   Lo - 95%   Hi - 95%
## ------------ ---------- ----------
##     0.74        0.50       1.10
## ----------------------------------
```

Dyspnea

```
with(data_final, ctable(vaccine_cov, dyspnea, prop = "r", useNA = "no", chisq = TRUE, OR=TRUE))
```

```
## Cross-Tabulation, Row Proportions
## vaccine_cov * dyspnea
## Data Frame: data_final
##
##
## ------------- --------- ------------- -------------- ---------------
##                 dyspnea            no            yes           Total
##   vaccine_cov
##            no             565 (29.5%)   1349 (70.5%)   1914 (100.0%)
##           yes              76 (43.9%)     97 (56.1%)    173 (100.0%)
##         Total             641 (30.7%)   1446 (69.3%)   2087 (100.0%)
## ------------- --------- ------------- -------------- ---------------
##
## ----------------------------
##  Chi.squared   df   p.value
## ------------- ---- ---------
##    14.8145     1     1e-04
## ----------------------------
##
## ----------------------------------
##  Odds Ratio   Lo - 95%   Hi - 95%
## ------------ ---------- ----------
##     0.53        0.39       0.73
## ----------------------------------
```

Respiratory discomfort

```
with(data_final, ctable(vaccine_cov, resp_disc, prop = "r", useNA = "no", chisq = TRUE, OR=TRUE))
```

```
## Cross-Tabulation, Row Proportions
```

```
## vaccine_cov * resp_disc
## Data Frame: data_final
##
##
## ------------- ----------- ------------- -------------- ---------------
##                 resp_disc            no            yes           Total
##   vaccine_cov
##            no               792 (43.5%)   1028 (56.5%)   1820 (100.0%)
##           yes                95 (55.2%)     77 (44.8%)    172 (100.0%)
##         Total               887 (44.5%)   1105 (55.5%)   1992 (100.0%)
## ------------- ----------- ------------- -------------- ---------------
##
## ----------------------------
##  Chi.squared   df   p.value
## ------------- ---- ---------
##    8.2652      1     0.004
## ----------------------------
##
## ----------------------------------
##  Odds Ratio   Lo - 95%   Hi - 95%
## ------------ ---------- ----------
##     0.62        0.46       0.86
## ----------------------------------
```

Desaturation

```
with(data_final, ctable(vaccine_cov, desaturation, prop = "r", useNA = "no", chisq = TRUE, OR=TRUE))
```

```
## Cross-Tabulation, Row Proportions
## vaccine_cov * desaturation
## Data Frame: data_final
##
##
## ------------- -------------- ------------- -------------- ---------------
##                 desaturation            no            yes           Total
##   vaccine_cov
##            no                  815 (44.3%)   1025 (55.7%)   1840 (100.0%)
##           yes                  103 (59.9%)     69 (40.1%)    172 (100.0%)
##         Total                  918 (45.6%)   1094 (54.4%)   2012 (100.0%)
## ------------- -------------- ------------- -------------- ---------------
##
## ----------------------------
##  Chi.squared   df   p.value
## ------------- ---- ---------
##    14.7886     1     1e-04
## ----------------------------
##
## ----------------------------------
##  Odds Ratio   Lo - 95%   Hi - 95%
## ------------ ---------- ----------
##     0.53        0.39       0.73
## ----------------------------------
```

Diarrhea

```r
with(data_final, ctable(vaccine_cov, diarrhea, prop = "r", useNA = "no", chisq = TRUE, OR=TRUE))
```

```
## Cross-Tabulation, Row Proportions
## vaccine_cov * diarrhea
## Data Frame: data_final
##
##
## ------------- ---------- -------------- ------------- ---------------
##                 diarrhea             no           yes           Total
##   vaccine_cov
##            no              1488 (88.7%)   189 (11.3%)   1677 (100.0%)
##           yes               143 (89.9%)    16 (10.1%)    159 (100.0%)
##         Total              1631 (88.8%)   205 (11.2%)   1836 (100.0%)
## ------------- ---------- -------------- ------------- ---------------
##
## ----------------------------
##  Chi.squared   df   p.value
## ------------- ---- ---------
##     0.109      1    0.7412
## ----------------------------
##
## ----------------------------------
##  Odds Ratio   Lo - 95%   Hi - 95%
## ------------ ---------- ----------
##     0.88        0.51       1.51
## ----------------------------------
```

Vomit

```r
with(data_final, ctable(vaccine_cov, vomit, prop = "r", useNA = "no", chisq = TRUE, OR=TRUE))
```

```
## Cross-Tabulation, Row Proportions
## vaccine_cov * vomit
## Data Frame: data_final
##
##
## ------------- ------- -------------- ------------- ---------------
##                 vomit             no           yes           Total
##   vaccine_cov
##            no           1480 (88.0%)   202 (12.0%)   1682 (100.0%)
##           yes            143 (89.9%)    16 (10.1%)    159 (100.0%)
##         Total           1623 (88.2%)   218 (11.8%)   1841 (100.0%)
## ------------- ------- -------------- ------------- ---------------
##
## ----------------------------
##  Chi.squared   df   p.value
## ------------- ---- ---------
##    0.3573      1     0.55
```

```
## ----------------------------
##
## ----------------------------------
##  Odds Ratio   Lo - 95%   Hi - 95%
## ------------ ---------- ----------
##     0.82        0.48       1.40
## ----------------------------------
```

Abdominal pain

```
with(data_final, ctable(vaccine_cov, abd_pain, prop = "r", useNA = "no", chisq = TRUE, OR=TRUE))
```

```
## Cross-Tabulation, Row Proportions
## vaccine_cov * abd_pain
## Data Frame: data_final
##
##
## ------------- ---------- -------------- ------------- ---------------
##                 abd_pain             no           yes           Total
##   vaccine_cov
##            no              1494 (90.1%)   164 ( 9.9%)   1658 (100.0%)
##           yes               138 (88.5%)    18 (11.5%)    156 (100.0%)
##         Total              1632 (90.0%)   182 (10.0%)   1814 (100.0%)
## ------------- ---------- -------------- ------------- ---------------
##
## ----------------------------
##  Chi.squared   df   p.value
## ------------- ---- ---------
##    0.2655      1    0.6064
## ----------------------------
##
## ----------------------------------
##  Odds Ratio   Lo - 95%   Hi - 95%
## ------------ ---------- ----------
##     1.19        0.71       1.99
## ----------------------------------
```

Fatigue

```
with(data_final, ctable(vaccine_cov, fatigue, prop = "r", useNA = "no", chisq = TRUE, OR=TRUE))
```

```
## Cross-Tabulation, Row Proportions
## vaccine_cov * fatigue
## Data Frame: data_final
##
##
## ------------- --------- -------------- ------------- ---------------
##                 fatigue             no           yes           Total
##   vaccine_cov
```

```
##            no              1147 (66.0%)   590 (34.0%)   1737 (100.0%)
##           yes               110 (67.5%)    53 (32.5%)    163 (100.0%)
##         Total              1257 (66.2%)   643 (33.8%)   1900 (100.0%)
## ------------- --------- -------------- ------------- ---------------
##
## ----------------------------
##  Chi.squared   df   p.value
## ------------- ---- ---------
##    0.0829      1    0.7735
## ----------------------------
##
## ----------------------------------
##  Odds Ratio   Lo - 95%   Hi - 95%
## ------------ ---------- ----------
##     0.94        0.67       1.32
## ----------------------------------
```

Olfactory loss

```
with(data_final, ctable(vaccine_cov, olfac_loss, prop = "r", useNA = "no", chisq = TRUE, OR=TRUE))
```

```
## Cross-Tabulation, Row Proportions
## vaccine_cov * olfac_loss
## Data Frame: data_final
##
##
## ------------- ------------ -------------- ------------- ---------------
##                 olfac_loss             no           yes           Total
##   vaccine_cov
##            no                 1372 (81.6%)   310 (18.4%)   1682 (100.0%)
##           yes                  127 (79.4%)    33 (20.6%)    160 (100.0%)
##         Total                 1499 (81.4%)   343 (18.6%)   1842 (100.0%)
## ------------- ------------ -------------- ------------- ---------------
##
## ----------------------------
##  Chi.squared   df   p.value
## ------------- ---- ---------
##    0.3308      1    0.5652
## ----------------------------
##
## ----------------------------------
##  Odds Ratio   Lo - 95%   Hi - 95%
## ------------ ---------- ----------
##     1.15        0.77       1.72
## ----------------------------------
```

Loss of taste

```
with(data_final, ctable(vaccine_cov, loss_taste, prop = "r", useNA = "no", chisq = TRUE, OR=TRUE))
```

```
## Cross-Tabulation, Row Proportions
## vaccine_cov * loss_taste
## Data Frame: data_final
##
##
## ------------- ------------ -------------- ------------- ---------------
##                 loss_taste             no           yes           Total
##   vaccine_cov
##            no               1403 (83.2%)   284 (16.8%)   1687 (100.0%)
##           yes                129 (81.1%)    30 (18.9%)    159 (100.0%)
##         Total               1532 (83.0%)   314 (17.0%)   1846 (100.0%)
## ------------- ------------ -------------- ------------- ---------------
##
## ----------------------------
##  Chi.squared   df   p.value
## ------------- ---- ---------
##    0.2937      1    0.5879
## ----------------------------
##
## ----------------------------------
##  Odds Ratio   Lo - 95%   Hi - 95%
## ------------ ---------- ----------
##     1.15        0.76       1.74
## ----------------------------------
```

Any respiratory symptom

```
with(data_final, ctable(vaccine_cov, resp_symp, prop = "r", useNA = "no", chisq = TRUE, OR=TRUE))
```

```
## Cross-Tabulation, Row Proportions
## vaccine_cov * resp_symp
## Data Frame: data_final
##
##
## ------------- ----------- ------------- -------------- ---------------
##                 resp_symp            no            yes           Total
##   vaccine_cov
##            no               361 (18.2%)   1618 (81.8%)   1979 (100.0%)
##           yes                56 (30.6%)    127 (69.4%)    183 (100.0%)
##         Total               417 (19.3%)   1745 (80.7%)   2162 (100.0%)
## ------------- ----------- ------------- -------------- ---------------
##
## ----------------------------
##  Chi.squared   df   p.value
## ------------- ---- ---------
##    15.6528     1     1e-04
## ----------------------------
##
## ----------------------------------
##  Odds Ratio   Lo - 95%   Hi - 95%
## ------------ ---------- ----------
##     0.51        0.36       0.71
```

```
## ----------------------------------
```

Any symptom

```
with(data_final, ctable(vaccine_cov, symptom, prop = "r", useNA = "no", chisq = TRUE, OR=TRUE))
```

```
## Cross-Tabulation, Row Proportions
## vaccine_cov * symptom
## Data Frame: data_final
##
##
## ------------- --------- ----------- -------------- ---------------
##                 symptom          no            yes           Total
##   vaccine_cov
##            no             78 (3.8%)   1980 (96.2%)   2058 (100.0%)
##           yes             16 (8.2%)    178 (91.8%)    194 (100.0%)
##         Total             94 (4.2%)   2158 (95.8%)   2252 (100.0%)
## ------------- --------- ----------- -------------- ---------------
##
## ----------------------------
##  Chi.squared   df   p.value
## ------------- ---- ---------
##     7.727      1    0.0054
## ----------------------------
##
## ----------------------------------
##  Odds Ratio   Lo - 95%   Hi - 95%
## ------------ ---------- ----------
##     0.44        0.25       0.77
## ----------------------------------
```

Outcome

```
# ICU
data_final <- data_final %>%
  mutate(icu = case_when(UTI == 1 ~ "yes",
                         UTI == 2 ~ "no",
                         TRUE ~ NA_character_))


# Length time in ICU
data_final<-  data_final%>%
  mutate(dt_enticu  = as.Date(DT_ENTUTI,  format = "%d/%m/%Y"),
         dt_exicu = as.Date(DT_SAIDUTI, format = "%d/%m/%Y"),
         time_icu = as.numeric(dt_exicu - dt_enticu)
  )

# ventilatory support
data_final <- data_final %>%
  mutate(ventilatory_support = case_when(SUPORT_VEN == 1 ~ "invasive",
```

```
                                SUPORT_VEN == 2 ~ "non-invasive",
                                  SUPORT_VEN == 3 ~ "no",
                                TRUE ~ NA_character_))

# Intubation
data_final <- data_final %>%
  mutate(intubation = case_when(SUPORT_VEN == 1 ~ "yes",
                                SUPORT_VEN == 2 | SUPORT_VEN == 3 ~ "no",
                                TRUE ~ NA_character_))
```

ICU

```
with(data_final, ctable(vaccine_cov, icu, prop = "r", useNA = "no", chisq = TRUE, OR=TRUE))
```

```
## Cross-Tabulation, Row Proportions
## vaccine_cov * icu
## Data Frame: data_final
##
##
## ------------- ----- -------------- ------------- ---------------
##                 icu             no           yes           Total
##   vaccine_cov
##            no         1239 (62.6%)   740 (37.4%)   1979 (100.0%)
##           yes          143 (76.5%)    44 (23.5%)    187 (100.0%)
##         Total         1382 (63.8%)   784 (36.2%)   2166 (100.0%)
## ------------- ----- -------------- ------------- ---------------
##
## ----------------------------
##  Chi.squared   df   p.value
## ------------- ---- ---------
##    13.6244     1     2e-04
## ----------------------------
##
## ----------------------------------
##  Odds Ratio   Lo - 95%   Hi - 95%
## ------------ ---------- ----------
##     0.52        0.36       0.73
## ----------------------------------
```

Length time in ICU

```
data_final_aux <- data_final %>%
  filter (icu == "yes")
```

```
datasummary((vaccine_cov) ~ time_icu*(n+media+DP+mediana+q25+q75+IQR),
            data = data_final_aux, output = 'markdown')
```

	n	media	DP	mediana	q25	q75	IQR
no	548.00	13.09	12.22	10.00	5.00	18.00	13.00
yes	21.00	9.38	9.30	5.00	3.00	13.00	10.00

```
#t-test
t.test(time_icu ~ vaccine_cov, data = data_final_aux)
```

```
##
##  Welch Two Sample t-test
##
## data:  time_icu by vaccine_cov
## t = 1.7692, df = 22.727, p-value = 0.09028
## alternative hypothesis: true difference in means between group no and group yes is not equal to 0
## 95 percent confidence interval:
##  -0.6305326  8.0474599
## sample estimates:
##  mean in group no mean in group yes
##         13.089416          9.380952
```

Ventilatory support

```
with(data_final, ctable(vaccine_cov, ventilatory_support, prop = "r", useNA = "no", chisq = TRUE, OR=TRU
```

```
## Cross-Tabulation, Row Proportions
## vaccine_cov * ventilatory_support
## Data Frame: data_final
##
##
## ------------- --------------------- ------------- ------------- -------------- ---------------
##                 ventilatory_support      invasive            no   non-invasive           Total
##   vaccine_cov
##            no                         368 (18.8%)   711 (36.3%)    880 (44.9%)   1959 (100.0%)
##           yes                           9 ( 4.8%)    99 (52.4%)     81 (42.9%)    189 (100.0%)
##         Total                         377 (17.6%)   810 (37.7%)    961 (44.7%)   2148 (100.0%)
## ------------- --------------------- ------------- ------------- -------------- ---------------
##
## ----------------------------
##  Chi.squared   df   p.value
## ------------- ---- ---------
##    31.3063     2       0
## ----------------------------
```

Intubation

```
with(data_final, ctable(vaccine_cov, intubation, prop = "r", useNA = "no", chisq = TRUE, OR=TRUE))
```

```
## Cross-Tabulation, Row Proportions
```

```
## vaccine_cov * intubation
## Data Frame: data_final
##
##
## ------------- ------------ -------------- ------------- ---------------
##                 intubation             no           yes           Total
##   vaccine_cov
##            no               1591 (81.2%)   368 (18.8%)   1959 (100.0%)
##           yes                180 (95.2%)     9 ( 4.8%)    189 (100.0%)
##         Total               1771 (82.4%)   377 (17.6%)   2148 (100.0%)
## ------------- ------------ -------------- ------------- ---------------
##
## ----------------------------
##  Chi.squared   df   p.value
## ------------- ---- ---------
##    22.4651     1       0
## ----------------------------
##
## ----------------------------------
##  Odds Ratio   Lo - 95%   Hi - 95%
## ------------ ---------- ----------
##     0.22        0.11       0.43
## ----------------------------------
```

Death

```
with(data_final, ctable(vaccine_cov, death, prop = "r", useNA = "no", chisq = TRUE, OR=TRUE))
```

```
## Cross-Tabulation, Row Proportions
## vaccine_cov * death
## Data Frame: data_final
##
##
## ------------- ------- -------------- ------------- ---------------
##                 death           cure         death           Total
##   vaccine_cov
##            no          1790 (85.9%)   294 (14.1%)   2084 (100.0%)
##           yes           194 (97.0%)     6 ( 3.0%)    200 (100.0%)
##         Total          1984 (86.9%)   300 (13.1%)   2284 (100.0%)
## ------------- ------- -------------- ------------- ---------------
##
## ----------------------------
##  Chi.squared   df   p.value
## ------------- ---- ---------
##    18.7715     1       0
## ----------------------------
##
## ----------------------------------
##  Odds Ratio   Lo - 95%   Hi - 95%
## ------------ ---------- ----------
##    0.188       0.083      0.428
## ----------------------------------
```

Propensity Scoring Method (PSM) - information about date of second dose

```
data_final <- data_final %>%
  mutate(vaccine1 = ifelse(vaccine_cov == "yes", 1, 0),
         id = 1:dim(data_final)[1])

data_final1 <- data_final %>%
  select(id, vaccine1, age, cardiac)

data_final1 <- data_final1 %>%
  mutate(
    cardiac1 = ifelse(is.na(cardiac) == TRUE, "na", cardiac)
  )

#PSM
psm1 <- matchit(vaccine1 ~ age + cardiac1, data = data_final1, method = "nearest", ratio =1)

summary(psm1)
```

```
##
## Call:
## matchit(formula = vaccine1 ~ age + cardiac1, data = data_final1,
##     method = "nearest", ratio = 1)
##
## Summary of Balance for All Data:
##              Means Treated Means Control Std. Mean Diff. Var. Ratio eCDF Mean
## distance            0.0965        0.0867          0.2648     1.9872    0.0595
## age                31.4400       29.7207          0.2227     1.1850    0.0409
## cardiac1na          0.5350        0.5960         -0.1222          .    0.0610
## cardiac1no          0.3550        0.3541          0.0018          .    0.0009
## cardiac1yes         0.1100        0.0499          0.1921          .    0.0601
##              eCDF Max
## distance       0.1179
## age            0.1056
## cardiac1na     0.0610
## cardiac1no     0.0009
## cardiac1yes    0.0601
##
##
## Summary of Balance for Matched Data:
##              Means Treated Means Control Std. Mean Diff. Var. Ratio eCDF Mean
## distance            0.0965        0.0965          0.0010     1.0023    0.0002
## age                31.4400       31.2950          0.0188     1.0431    0.0035
## cardiac1na          0.5350        0.5300          0.0100          .    0.0050
## cardiac1no          0.3550        0.3550          0.0000          .    0.0000
## cardiac1yes         0.1100        0.1150         -0.0160          .    0.0050
##              eCDF Max Std. Pair Dist.
## distance        0.010          0.0042
## age             0.015          0.0214
## cardiac1na      0.005          0.0100
## cardiac1no      0.000          0.0000
```

```
## cardiac1yes    0.005          0.0160
##
## Percent Balance Improvement:
##             Std. Mean Diff. Var. Ratio eCDF Mean eCDF Max
## distance                99.6       99.7      99.6     91.5
## age                     91.6       75.2      91.6     85.8
## cardiac1na              91.8          .      91.8     91.8
## cardiac1no             100.0          .     100.0    100.0
## cardiac1yes             91.7          .      91.7     91.7
##
## Sample Sizes:
##           Control Treated
## All          2084     200
## Matched       200     200
## Unmatched    1884       0
## Discarded       0       0
```

```
plot(psm1, type = "jitter", interactive = FALSE)
```

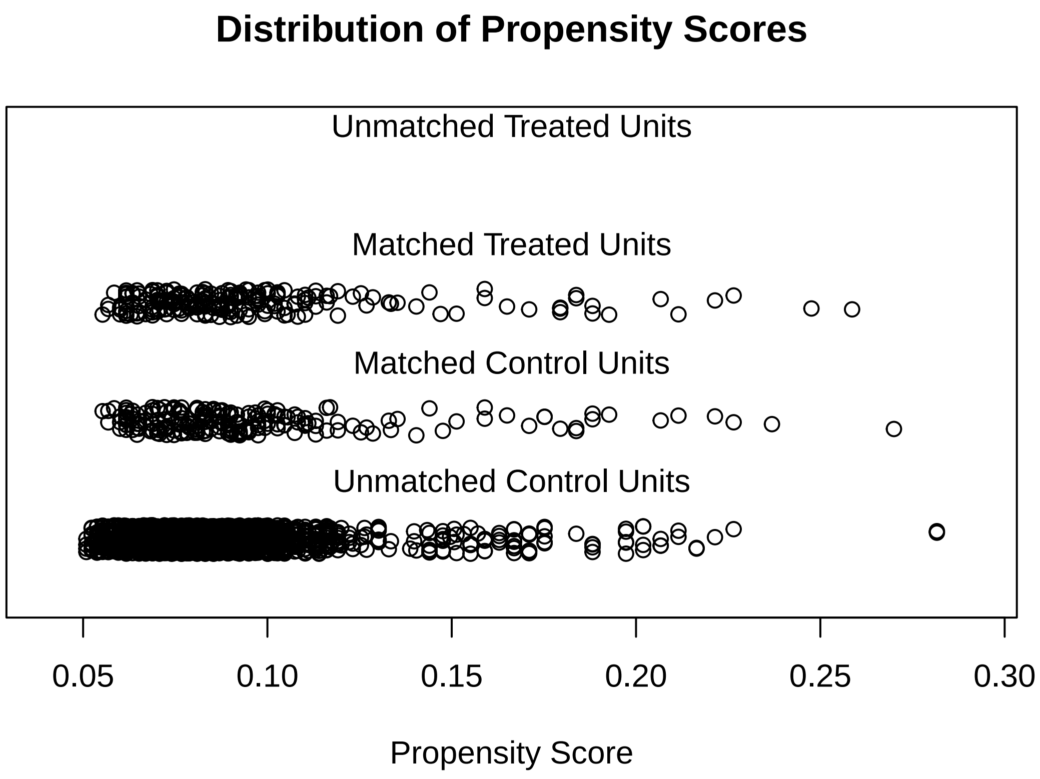


```
plot(psm1, type = "qq", interactive = FALSE,
     which.xs = c("age", "cardiac1"))
```

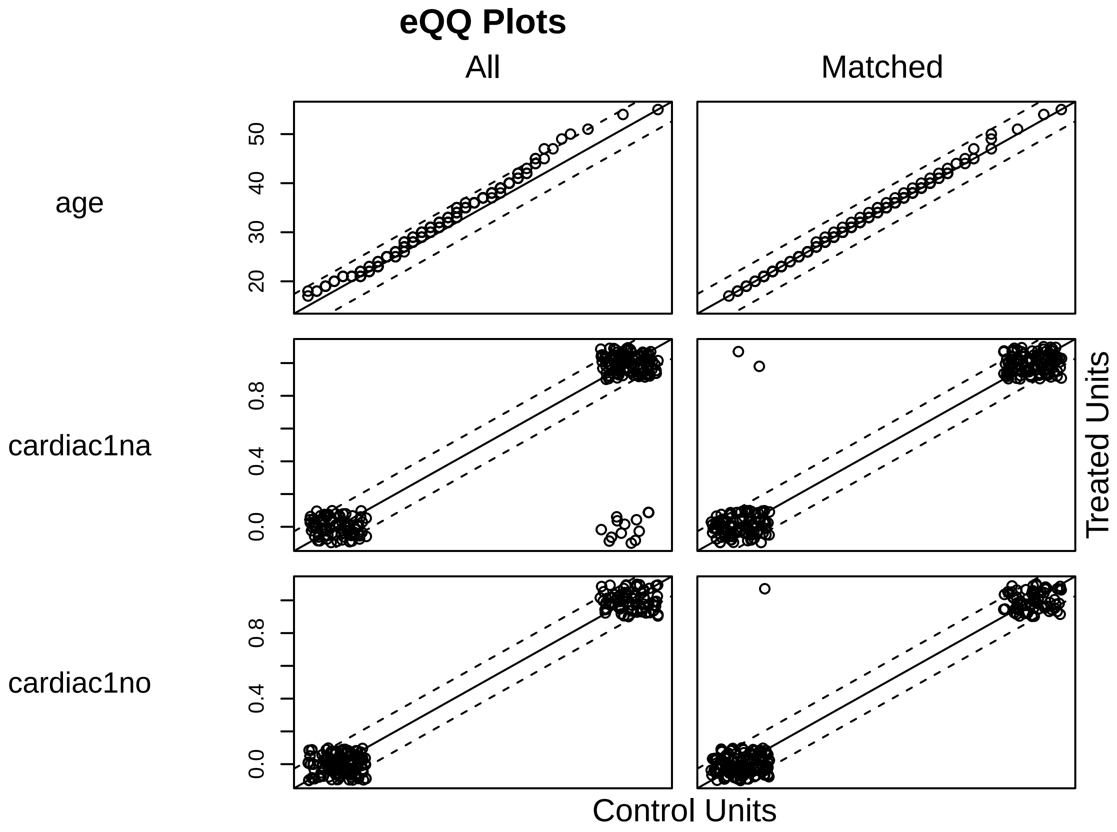

eQQ Plots
All
Matched
age
cardiac1na
cardiac1no
Treated Units
Control Units
20
30
40
50
0.0
0.4
0.8
0.0
0.4
0.8

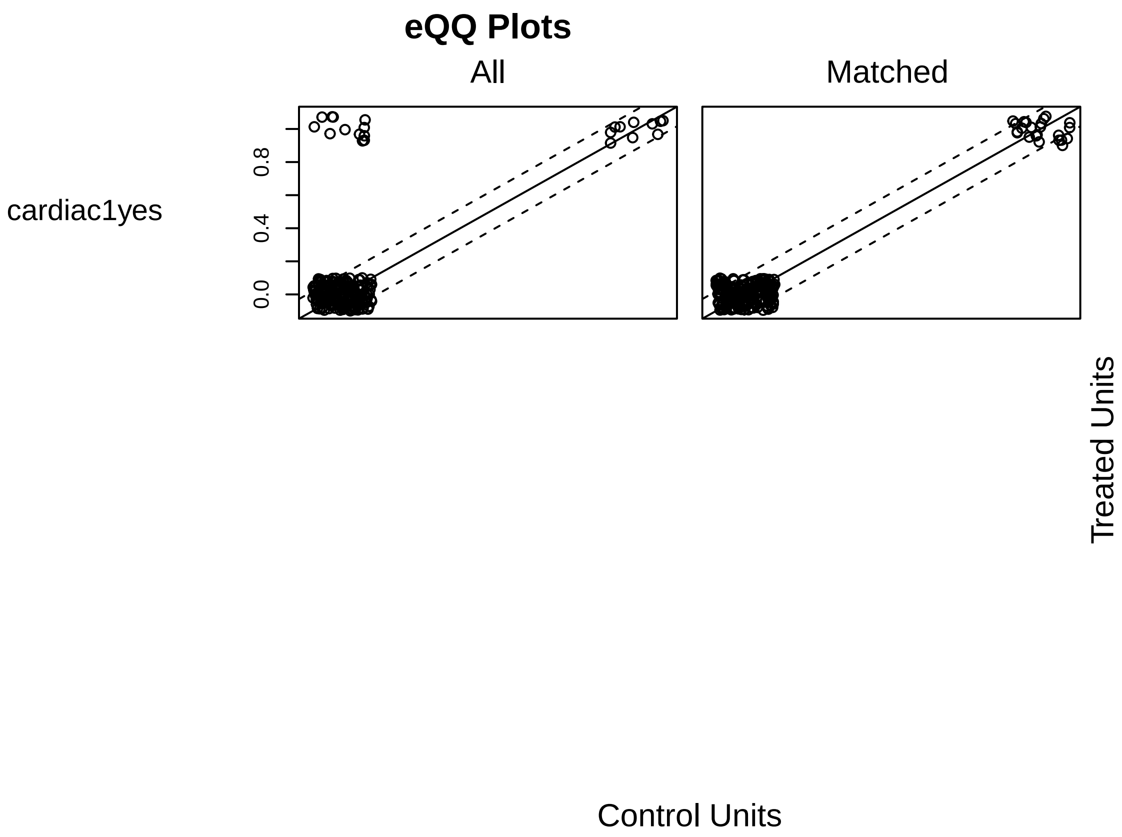


```
plot(summary(psm1))
```

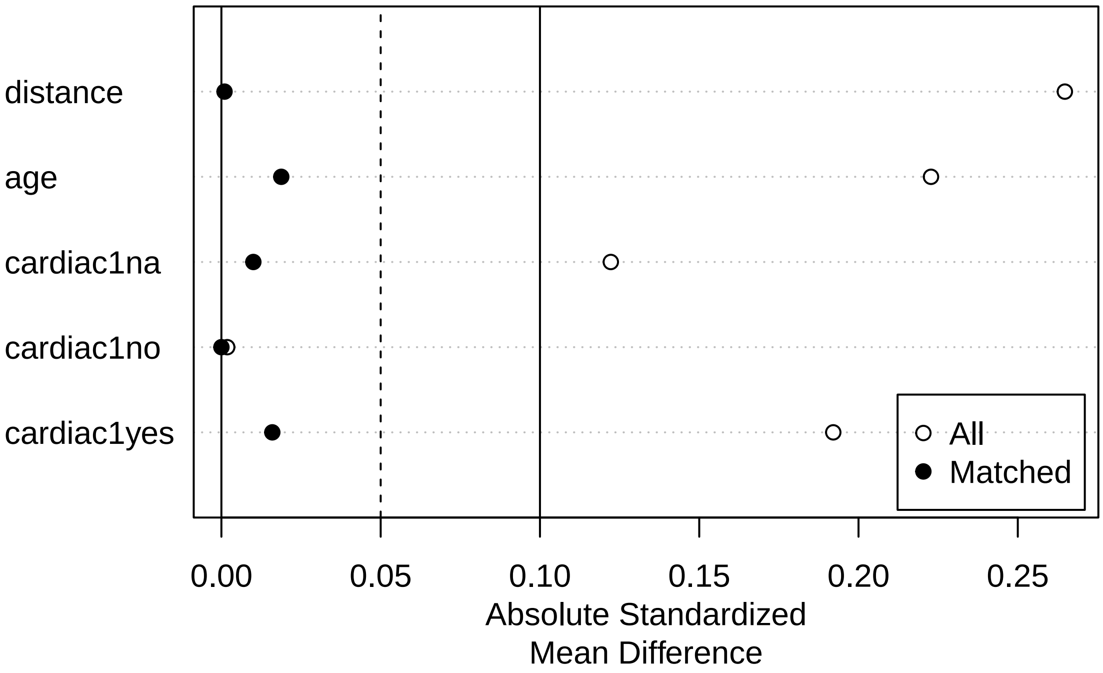


```
#Selecting only the selected observations
data_aux <- data_final1[psm1$weights==1, ]

#Now let's join data_aux with data_final

data_psm <- right_join(data_final, data_aux, by= c("id", "vaccine1", "age", "cardiac"))

freq(data_psm$vaccine_cov)
```

```
##       n  % val%
## no  200 50   50
## yes 200 50   50
```

For outcomes

ICU

```
with(data_psm, ctable(vaccine_cov, icu, prop = "r", useNA = "no", chisq = TRUE, OR=TRUE))
```

```
## Cross-Tabulation, Row Proportions
## vaccine_cov * icu
## Data Frame: data_psm
##
```

```
##
## ------------- ----- ------------- ------------- --------------
##                 icu            no           yes          Total
##   vaccine_cov
##            no         121 (63.7%)    69 (36.3%)   190 (100.0%)
##           yes         143 (76.5%)    44 (23.5%)   187 (100.0%)
##         Total         264 (70.0%)   113 (30.0%)   377 (100.0%)
## ------------- ----- ------------- ------------- --------------
##
## ----------------------------
##  Chi.squared   df   p.value
## ------------- ---- ---------
##    6.7444      1    0.0094
## ----------------------------
##
## ----------------------------------
##  Odds Ratio   Lo - 95%   Hi - 95%
## ------------ ---------- ----------
##     0.54        0.34       0.85
## ----------------------------------
```

Length time in ICU

```
data_psm_aux <- data_psm %>%
  filter (icu == "yes")
```

```
datasummary((vaccine_cov) ~ time_icu*(n+media+DP+mediana+q25+q75+IQR),
            data = data_psm_aux, output = 'markdown')
```

	n	media	DP	mediana	q25	q75	IQR
no	57.00	14.09	11.67	12.00	5.00	18.00	13.00
yes	21.00	9.38	9.30	5.00	3.00	13.00	10.00

```
#teste t
t.test(time_icu ~ vaccine_cov, data = data_psm_aux)
```

```
##
##  Welch Two Sample t-test
##
## data:  time_icu by vaccine_cov
## t = 1.8447, df = 44.556, p-value = 0.07173
## alternative hypothesis: true difference in means between group no and group yes is not equal to 0
## 95 percent confidence interval:
##  -0.4335558  9.8470897
## sample estimates:
##  mean in group no mean in group yes
##         14.087719          9.380952
```

Ventilatory support

```
with(data_psm, ctable(vaccine_cov, ventilatory_support, prop = "r", useNA = "no", chisq = TRUE, OR=TRUE
```

```
## Cross-Tabulation, Row Proportions
## vaccine_cov * ventilatory_support
## Data Frame: data_psm
##
##
## ------------- --------------------- ------------ ------------- -------------- --------------
##                 ventilatory_support     invasive            no   non-invasive          Total
##   vaccine_cov
##            no                         39 (20.6%)    68 (36.0%)     82 (43.4%)   189 (100.0%)
##           yes                          9 ( 4.8%)    99 (52.4%)     81 (42.9%)   189 (100.0%)
##         Total                         48 (12.7%)   167 (44.2%)    163 (43.1%)   378 (100.0%)
## ------------- --------------------- ------------ ------------- -------------- --------------
##
## ----------------------------
##  Chi.squared   df   p.value
## ------------- ---- ---------
##    24.5106     2       0
## ----------------------------
```

Intubation

```
with(data_psm, ctable(vaccine_cov, intubation, prop = "r", useNA = "no", chisq = TRUE, OR=TRUE))
```

```
## Cross-Tabulation, Row Proportions
## vaccine_cov * intubation
## Data Frame: data_psm
##
##
## ------------- ------------ ------------- ------------ --------------
##                 intubation            no          yes          Total
##   vaccine_cov
##            no                150 (79.4%)   39 (20.6%)   189 (100.0%)
##           yes                180 (95.2%)    9 ( 4.8%)   189 (100.0%)
##         Total                330 (87.3%)   48 (12.7%)   378 (100.0%)
## ------------- ------------ ------------- ------------ --------------
##
## ----------------------------
##  Chi.squared   df   p.value
## ------------- ---- ---------
##    20.0693     1       0
## ----------------------------
##
## ----------------------------------
##  Odds Ratio   Lo - 95%   Hi - 95%
## ------------ ---------- ----------
##     0.19        0.09       0.41
## ----------------------------------
```

Death

```
with(data_psm, ctable(vaccine_cov, death, prop = "r", useNA = "no", chisq = TRUE, OR=TRUE))
```

```
## Cross-Tabulation, Row Proportions
## vaccine_cov * death
## Data Frame: data_psm
##
##
## ------------- ------- ------------- ------------ --------------
##                 death          cure        death          Total
##   vaccine_cov
##            no           174 (87.0%)   26 (13.0%)   200 (100.0%)
##           yes           194 (97.0%)    6 ( 3.0%)   200 (100.0%)
##         Total           368 (92.0%)   32 ( 8.0%)   400 (100.0%)
## ------------- ------- ------------- ------------ --------------
##
## ----------------------------
##  Chi.squared   df   p.value
## ------------- ---- ---------
##    12.2622     1     5e-04
## ----------------------------
##
## ----------------------------------
##  Odds Ratio   Lo - 95%   Hi - 95%
## ------------ ---------- ----------
##    0.207       0.083      0.515
## ----------------------------------
```

Multivariate Imputation by Chained Equations (MICE)

A total of 2284 patients were analyzed and divided into two groups, according to their COVID-19 vaccination status: unvaccinated (n = 2084; 91.2%) and vaccinated (n = 200; 8.8%) groups. With respect to this dataset, to deal with possible nonresponse bias, we explore the missing values and we conduct a multiple imputation.

```
data_na <- data_final %>%
  select(
    vaccine_cov,
    age,
    white_color,
    cardiac,
    diabetes,
    hematologic,
    obesity,
    asthma,
    hepatic,
    neurologic,
    pneumologic,
    imuno,
    renal,
    education2,
```

```r
    residence2,
    fever,
    cough,
    sore_throat,
    dyspnea,
    resp_disc,
    desaturation,
    diarrhea,
    vomit,
    abd_pain,
    fatigue,
    olfac_loss,
    loss_taste,
    icu,
    intubation,
    death
  )

data_na$vaccine_cov <- as.factor(data_na$vaccine_cov)
data_na$white_color <- as.factor(data_na$white_color)
data_na$cardiac <- as.factor(data_na$cardiac)
data_na$diabetes <- as.factor(data_na$diabetes)
data_na$hematologic <- as.factor(data_na$hematologic)
data_na$obesity <- as.factor(data_na$obesity)
data_na$asthma <- as.factor(data_na$asthma)
data_na$hepatic <- as.factor(data_na$hepatic)
data_na$neurologic <- as.factor(data_na$neurologic)
data_na$pneumologic <- as.factor(data_na$pneumologic)
data_na$imuno <- as.factor(data_na$imuno)
data_na$renal <- as.factor(data_na$renal)
data_na$residence2 <- as.factor(data_na$residence2)
data_na$fever <- as.factor(data_na$fever)
data_na$cough <- as.factor(data_na$cough)
data_na$sore_throat <- as.factor(data_na$sore_throat)
data_na$dyspnea <- as.factor(data_na$dyspnea)
data_na$resp_disc <- as.factor(data_na$resp_disc)
data_na$desaturation <- as.factor(data_na$desaturation)
data_na$diarrhea <- as.factor(data_na$diarrhea)
data_na$vomit <- as.factor(data_na$vomit)
data_na$abd_pain <- as.factor(data_na$abd_pain)
data_na$fatigue <- as.factor(data_na$fatigue)
data_na$olfac_loss <- as.factor(data_na$olfac_loss)
data_na$loss_taste <- as.factor(data_na$loss_taste)
data_na$icu <- as.factor(data_na$icu)
data_na$intubation <- as.factor(data_na$intubation)
data_na$death <- as.factor(data_na$death)

print(dfSummary(data_na, varnumbers = FALSE), method = "render")
```

Note that all comorbities variables and the education variable have high proportions of missing data. The percentage of valid responses is, at least, of 79,4% for variables related to the symptoms.

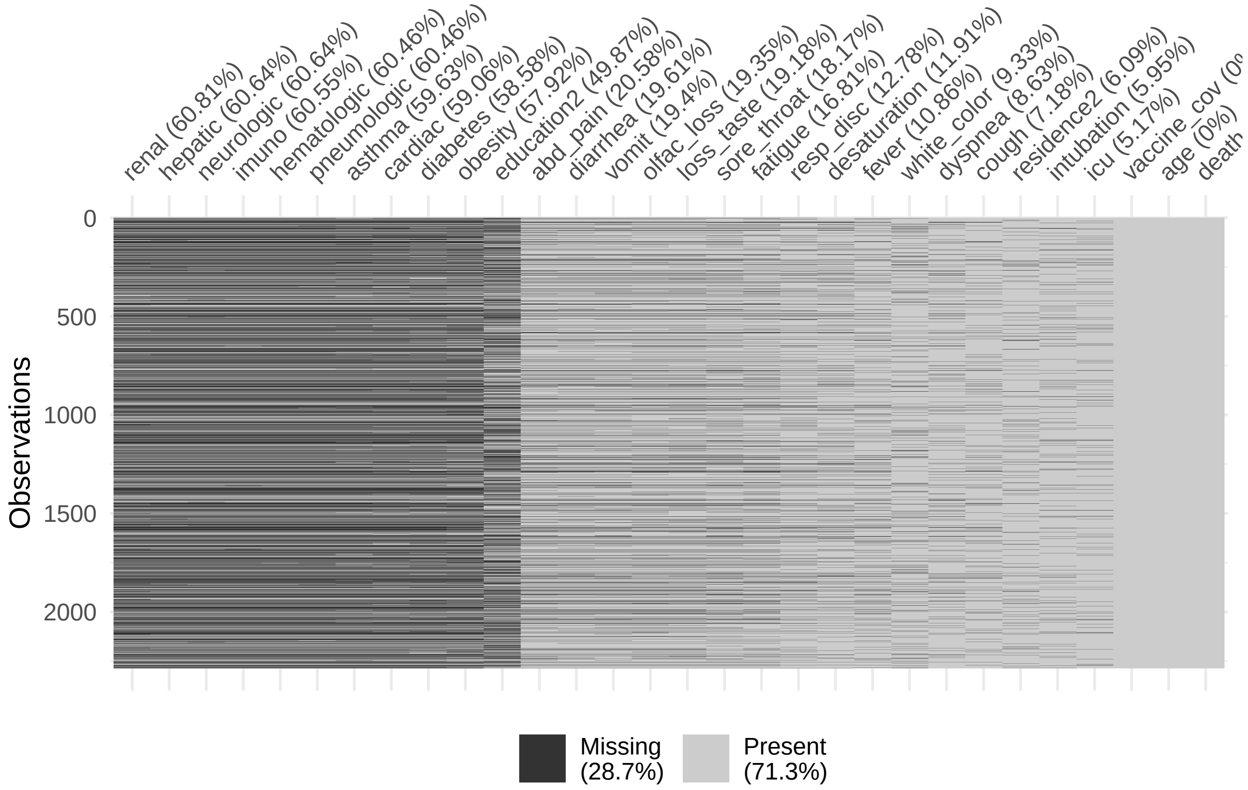

To deal with missing data, we consider a multiple imputation by fully conditional specification. The mice package implements this method, where each incomplete variable is imputed by a separate model. According to the previously analysis, the most of the variables with missing data are binary. The variables “hematologic”, “hepatic”, “neurologic”, “pneumologic”, “renal” and “imuno” present categories extremely unbalanced, with approximately 60% missing data each one. For this reason and to avoid problems of bias and efficiency, these variables will not be considered in the process of multiple imputation. The “residence” variable was recategorized in two levels, urban/periurban and rural because the periurban level has only 6 observations. We considered as a imputation method for dichotomous variable the logistic regression (logreg), except when the percentage of missing values for the variable is greater than 50%. In this situation, we consider a a bootstrapped logistic regression model (logreg.boot). For education variable, we considered a proportional odds model (polr). Following, because of high percentage of missing data on some variables, we present 100 imputed datasets e the trace plots to investigate the convergence of the method.

```
cols.dont.want <- c("hematologic", "hepatic", "neurologic", "pneumologic", "renal", "imuno")
data_na2 <- data_na[, ! names(data_na) %in% cols.dont.want, drop = F]

mice.impute.logreg <- function (y, ry, x, wy = NULL, ...)
{
  if (is.null(wy))
    wy <- !ry
  aug <- augment(y, ry, x, wy)
  x <- aug$x
  y <- aug$y
  ry <- aug$ry
  wy <- aug$wy
  w <- aug$w
```

```r
  x <- cbind(1, as.matrix(x))
  expr <- expression(glm.fit(x = x[ry, , drop = FALSE], y = y[ry],
                             family = binomial(link = logit), weights = w[ry], maxit = 150))
  fit <- eval(expr)
  fit.sum <- summary.glm(fit)
  beta <- coef(fit)
  rv <- t(chol(sym(fit.sum$cov.unscaled)))
  beta.star <- beta + rv %*% rnorm(ncol(rv))
  p <- 1/(1 + exp(-(x[wy, , drop = FALSE] %*% beta.star)))
  vec <- (runif(nrow(p)) <= p)
  vec[vec] <- 1
  if (is.factor(y)) {
    vec <- factor(vec, c(0, 1), levels(y))
  }
  vec
}

imputed_Data <- mice(data_na2, m=100, maxit = 60, method = c("", "", "logreg", rep("logreg.boot", 4), "
                          rep("logreg", 16)), printFlag = FALSE, seed = 300)

summary(imputed_Data)
```

```
## Class: mids
## Number of multiple imputations:  100
## Imputation methods:
##   vaccine_cov           age   white_color       cardiac      diabetes
##            ""            ""      "logreg" "logreg.boot" "logreg.boot"
##       obesity        asthma    education2    residence2         fever
## "logreg.boot" "logreg.boot"     "polyreg"      "logreg"      "logreg"
##         cough   sore_throat       dyspnea     resp_disc   desaturation
##      "logreg"      "logreg"      "logreg"      "logreg"      "logreg"
##      diarrhea         vomit      abd_pain       fatigue    olfac_loss
##      "logreg"      "logreg"      "logreg"      "logreg"      "logreg"
##    loss_taste           icu    intubation         death
##      "logreg"      "logreg"      "logreg"            ""
## PredictorMatrix:
##             vaccine_cov age white_color cardiac diabetes obesity asthma
## vaccine_cov           0   1           1       1        1       1      1
## age                   1   0           1       1        1       1      1
## white_color           1   1           0       1        1       1      1
## cardiac               1   1           1       0        1       1      1
## diabetes              1   1           1       1        0       1      1
## obesity               1   1           1       1        1       0      1
##             education2 residence2 fever cough sore_throat dyspnea resp_disc
## vaccine_cov          1          1     1     1           1       1         1
## age                  1          1     1     1           1       1         1
## white_color          1          1     1     1           1       1         1
## cardiac              1          1     1     1           1       1         1
## diabetes             1          1     1     1           1       1         1
## obesity              1          1     1     1           1       1         1
##             desaturation diarrhea vomit abd_pain fatigue olfac_loss loss_taste
## vaccine_cov            1        1     1        1       1          1          1
## age                    1        1     1        1       1          1          1
```

```
## white_color             1          1      1          1          1             1             1
## cardiac                 1          1      1          1          1             1             1
## diabetes                1          1      1          1          1             1             1
## obesity                 1          1      1          1          1             1             1
##              icu intubation death
## vaccine_cov    1           1     1
## age            1           1     1
## white_color    1           1     1
## cardiac        1           1     1
## diabetes       1           1     1
## obesity        1           1     1
```

```
plot(imputed_Data)
```

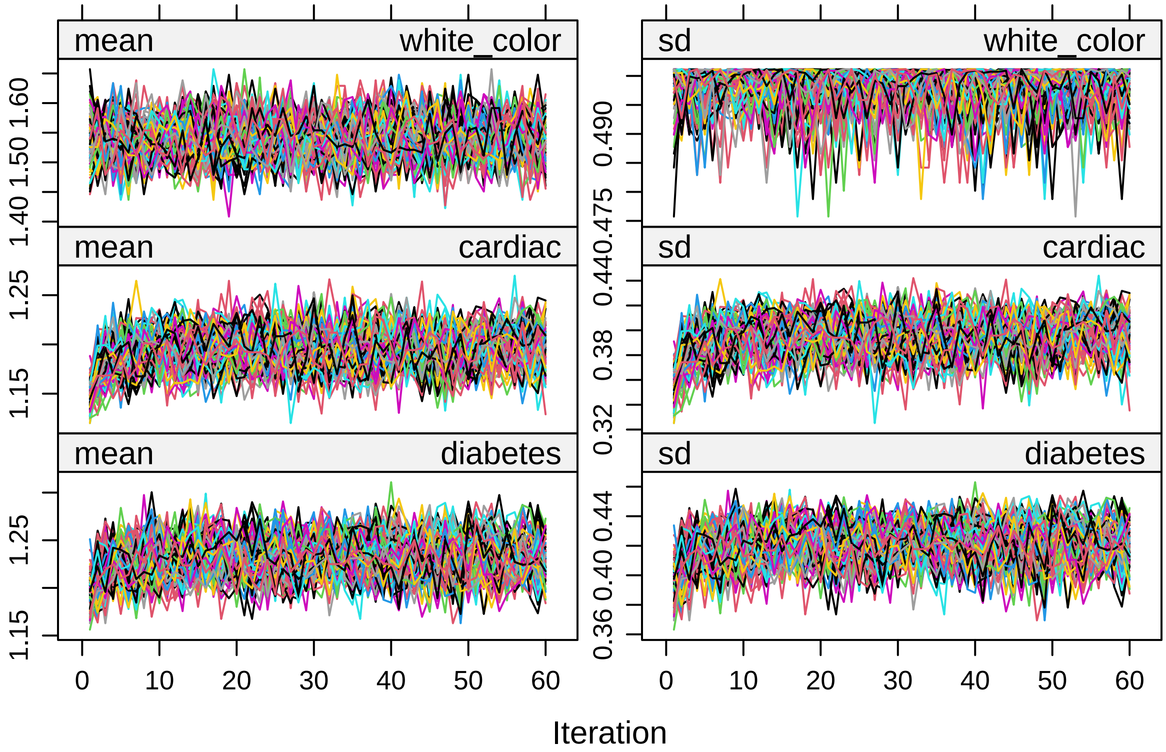

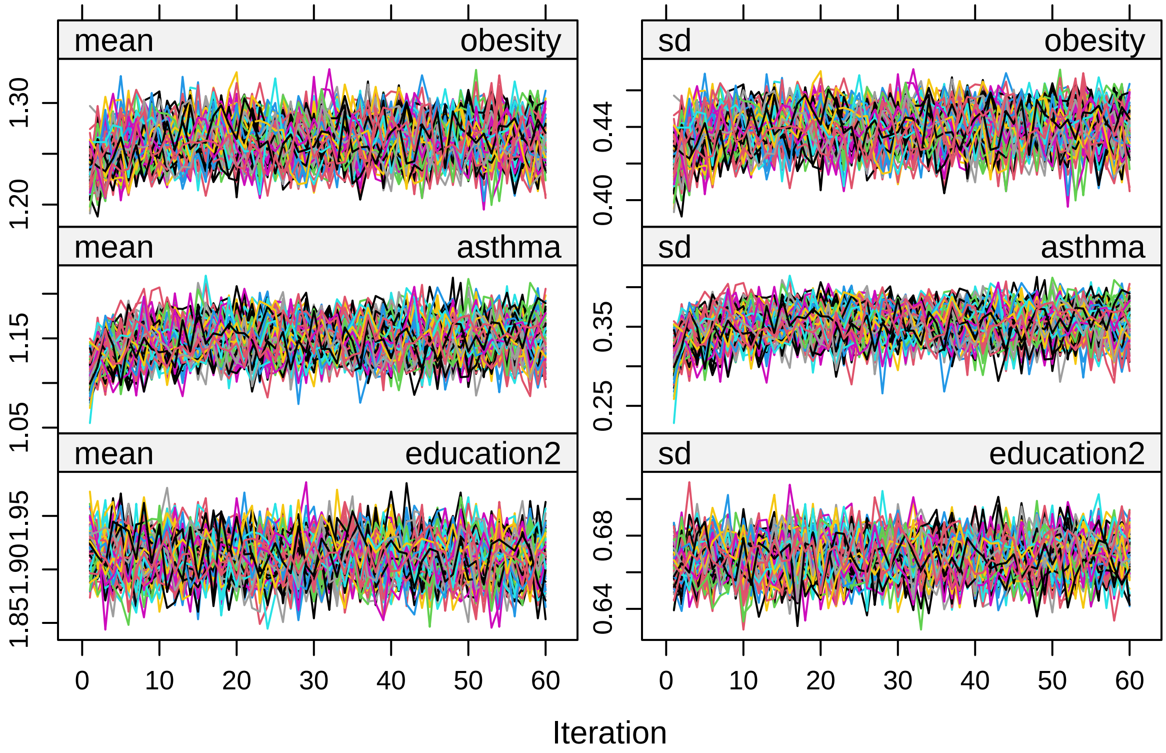
mean
obesity
sd
obesity
mean
asthma
sd
asthma
mean
education2
sd
education2
1.20
1.30
0.40
0.44
1.05
1.15
0.25
0.35
1.85
1.90
1.95
0.64
0.68
0
10
20
30
40
50
60
Iteration

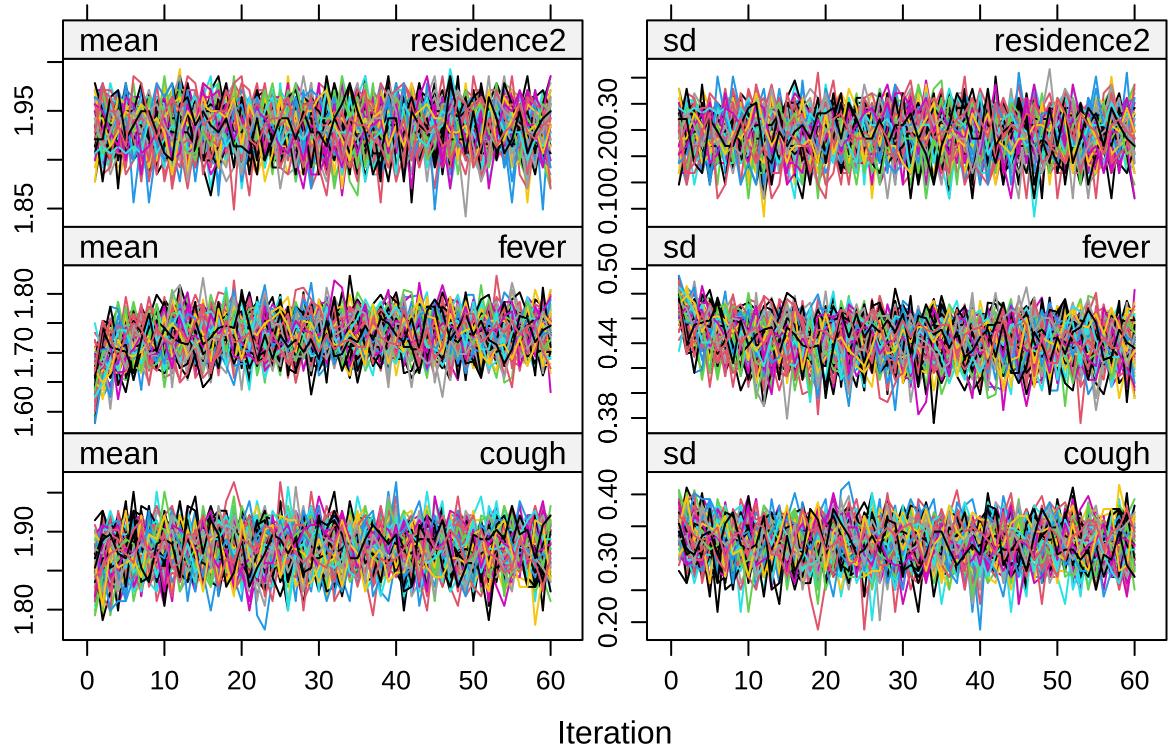
mean
residence2
sd
residence2
mean
fever
sd
fever
mean
cough
sd
cough
Iteration

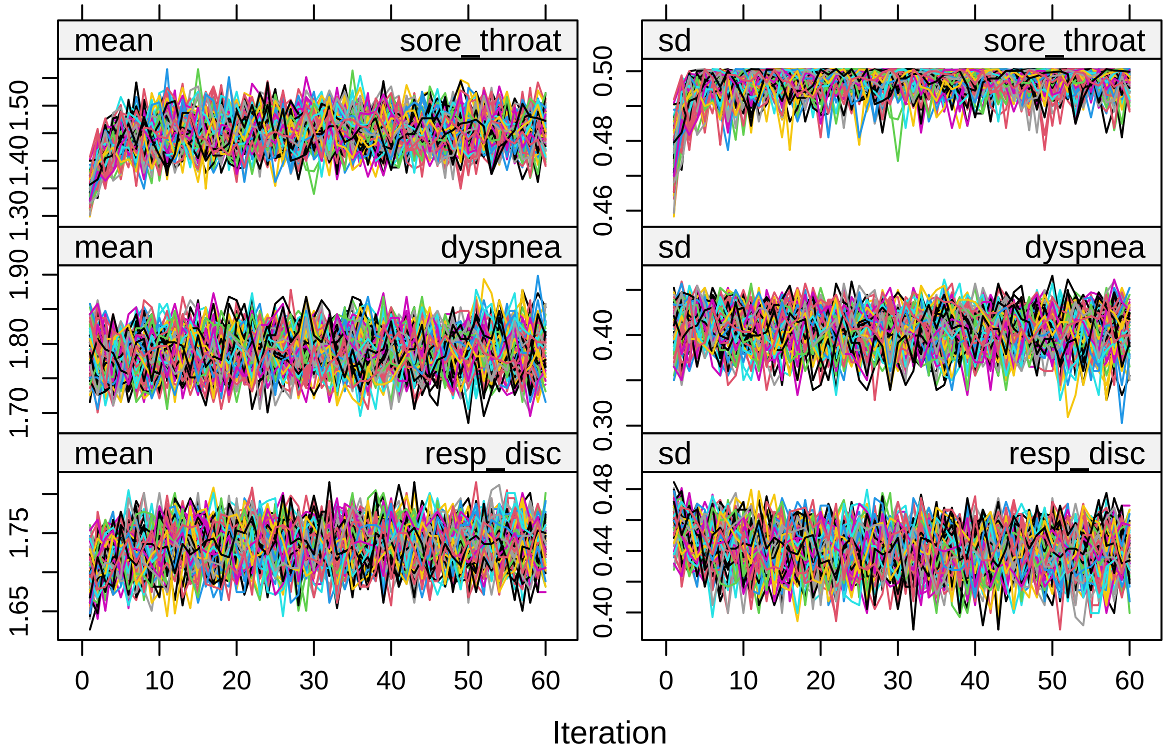

mean
sore_throat
sd
sore_throat
mean
dyspnea
sd
dyspnea
mean
resp_disc
sd
resp_disc
1.30
1.40
1.50
1.70
1.80
1.90
1.65
1.75
0.46
0.48
0.50
0.30
0.40
0.40
0.44
0.48
0
10
20
30
40
50
60
Iteration

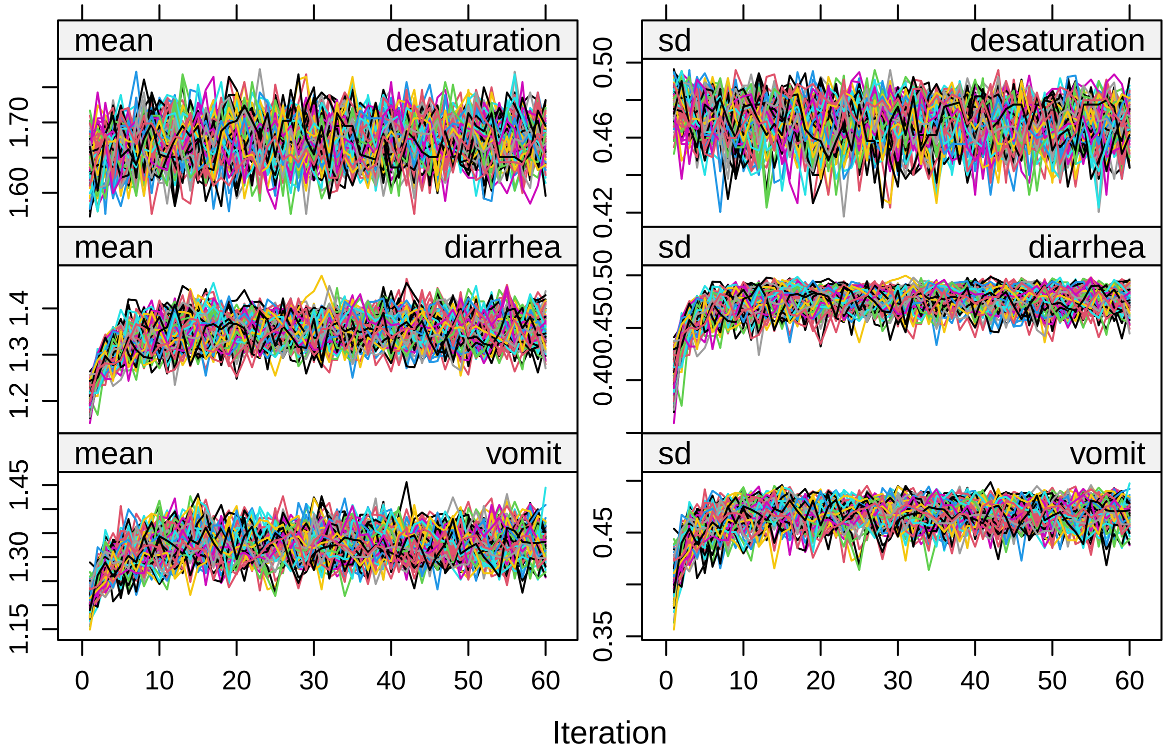
mean desaturation
mean diarrhea
mean vomit
sd desaturation
sd diarrhea
sd vomit
1.60
1.70
1.2 1.3 1.4
1.15
1.30
1.45
0.42
0.46
0.50
0.400.450.50
0.35
0.45
0
10
20
30
40
50
60
Iteration

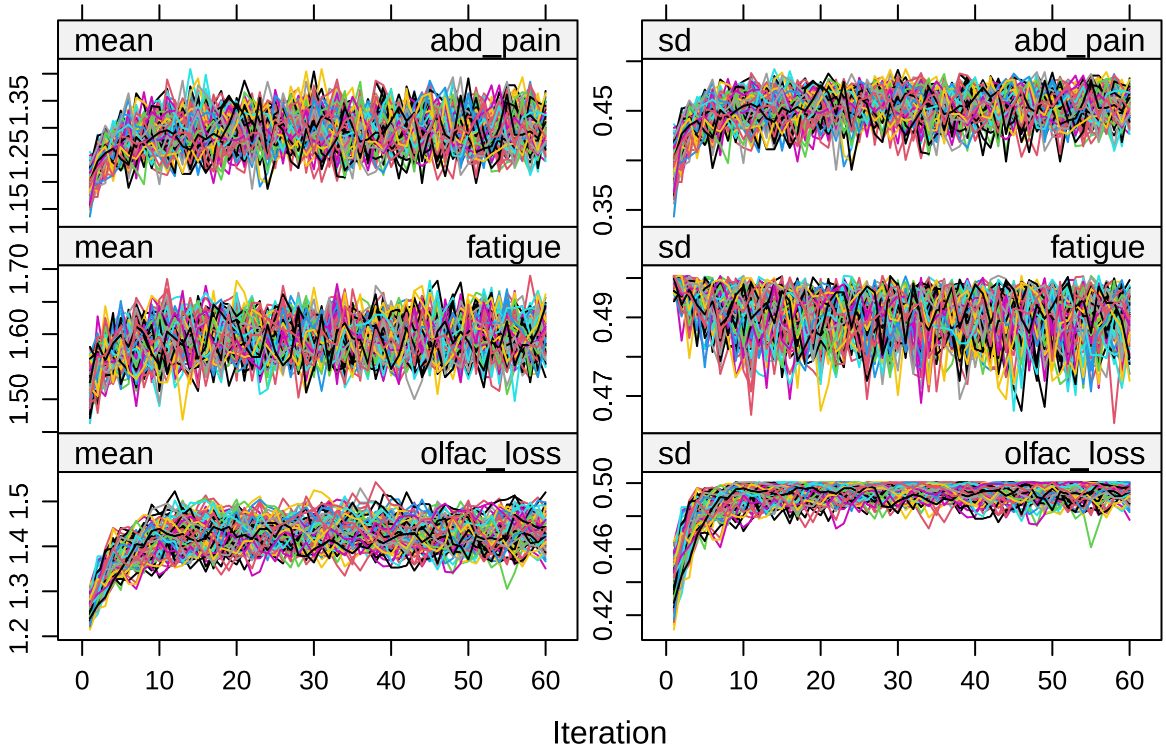

mean
abd_pain
mean
fatigue
mean
olfac_loss
sd
abd_pain
sd
fatigue
sd
olfac_loss
1.15
1.25
1.35
1.50
1.60
1.70
1.2
1.3
1.4
1.5
0.35
0.45
0.47
0.49
0.42
0.46
0.50
0
10
20
30
40
50
60
Iteration

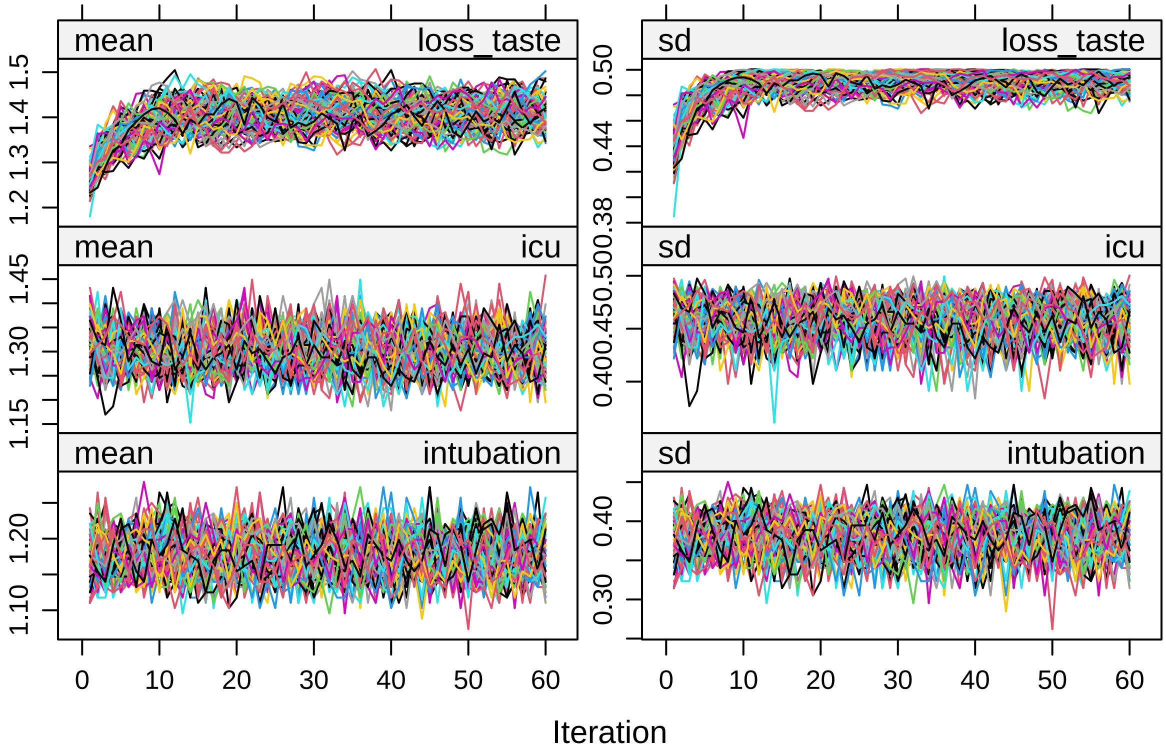


```
dat_imp <- mids2milist(imputed_Data)
```

Each one of these 100 imputed datasets were analysed using the function `with()`, and including an expression for the statistical analysis approach. Procedures to pool Chi-square values are available in the miceadds package. To combine the 100 odds ratios, we use the miceafter package.

Baseline characteristics of the subjects according to vaccination status

White color

```
fit_w <- with(dat_imp, exp = chisq.test(vaccine_cov, white_color))
Stat_w <- numeric()
for (i in 1:imputed_Data$m) Stat_w[i] <- fit_w$statistics[[i]]$statistic
micombine.chisquare(Stat_w, 1, display = TRUE, version=1)
```

```
## Combination of Chi Square Statistics for Multiply Imputed Data
## Using 100 Imputed Data Sets
## F(1, 18484.24)=3.054     p=0.08054
```

Cardiac

```r
fit_c <- with(dat_imp, exp = chisq.test(vaccine_cov, cardiac))
Stat_c <- numeric()
for (i in 1:imputed_Data$m) Stat_c[i] <- fit_c$statistics[[i]]$statistic
micombine.chisquare(Stat_c, 1, display = TRUE, version=1)
```

```
## Combination of Chi Square Statistics for Multiply Imputed Data
## Using 100 Imputed Data Sets
## F(1, 270.94)=4.983     p=0.02642
```

Diabetes

```r
fit_d <- with(dat_imp, exp = chisq.test(vaccine_cov, diabetes))
Stat_d <- numeric()
for (i in 1:imputed_Data$m) Stat_d[i] <- fit_d$statistics[[i]]$statistic
micombine.chisquare(Stat_d, 1, display = TRUE, version=1)
```

```
## Combination of Chi Square Statistics for Multiply Imputed Data
## Using 100 Imputed Data Sets
## F(1, 394.63)=0.892     p=0.34539
```

Obesity

```r
fit_o <- with(dat_imp, exp = chisq.test(vaccine_cov, obesity))
Stat_o <- numeric()
for (i in 1:imputed_Data$m) Stat_o[i] <- fit_o$statistics[[i]]$statistic
micombine.chisquare(Stat_o, 1, display = TRUE, version=1)
```

```
## Combination of Chi Square Statistics for Multiply Imputed Data
## Using 100 Imputed Data Sets
## F(1, 439.29)=1.475     p=0.22518
```

Asthma

```r
fit_a <- with(dat_imp, exp = chisq.test(vaccine_cov, asthma))
Stat_a <- numeric()
for (i in 1:imputed_Data$m) Stat_a[i] <- fit_a$statistics[[i]]$statistic
micombine.chisquare(Stat_a, 1, display = TRUE, version=1)
```

```
## Combination of Chi Square Statistics for Multiply Imputed Data
## Using 100 Imputed Data Sets
## F(1, 273.82)=1.085     p=0.29841
```

Education

```r
fit_e <- with(dat_imp, exp = chisq.test(vaccine_cov, education2))
Stat_e <- numeric()
for (i in 1:imputed_Data$m) Stat_e[i] <- fit_e$statistics[[i]]$statistic
micombine.chisquare(Stat_e, 2, display = TRUE, version=1)
```

```
## Combination of Chi Square Statistics for Multiply Imputed Data
## Using 100 Imputed Data Sets
## F(2, 755.7)=2.461     p=0.08601
```

Residence area 2 (grouping the categories urban and periurban)

```r
fit_res <- with(dat_imp, exp = chisq.test(vaccine_cov, residence2))
Stat_res <- numeric()
for (i in 1:imputed_Data$m) Stat_res[i] <- fit_res$statistics[[i]]$statistic
micombine.chisquare(Stat_res, 2, display = TRUE, version=1)
```

```
## Combination of Chi Square Statistics for Multiply Imputed Data
## Using 100 Imputed Data Sets
## F(2, 44650.47)=0.07     p=0.93195
```

Characteristics of COVID-19 symptoms by vaccination status

Fever

```r
pool_odds_ratio(with(dat_imp, expr=odds_ratio(vaccine_cov ~ fever)))
```

```
##      pooled OR 95 CI low 95 CI high
## [1,] 0.6434665 0.4735422  0.8743657
## attr(,"class")
## [1] "mipool"
```

Cough

```r
pool_odds_ratio(with(dat_imp, expr=odds_ratio(vaccine_cov ~ cough)))
```

```
##      pooled OR 95 CI low 95 CI high
## [1,] 0.8208345 0.5776051   1.166488
## attr(,"class")
## [1] "mipool"
```

Sore throat

```r
pool_odds_ratio(with(dat_imp, expr=odds_ratio(vaccine_cov ~ sore_throat)))
```

```
##      pooled OR 95 CI low 95 CI high
## [1,] 0.7729552 0.5312457   1.124639
## attr(,"class")
## [1] "mipool"
```

Dyspnea

```r
pool_odds_ratio(with(dat_imp, expr=odds_ratio(vaccine_cov ~ dyspnea)))
```

```
##      pooled OR 95 CI low 95 CI high
## [1,]  0.562676  0.412656  0.7672355
## attr(,"class")
## [1] "mipool"
```

Respiratory discomfort

```r
pool_odds_ratio(with(dat_imp, expr=odds_ratio(vaccine_cov ~ resp_disc)))
```

```
##      pooled OR 95 CI low 95 CI high
## [1,] 0.6371953 0.4682167   0.867158
## attr(,"class")
## [1] "mipool"
```

Desaturation

```r
pool_odds_ratio(with(dat_imp, expr=odds_ratio(vaccine_cov ~ desaturation)))
```

```
##      pooled OR 95 CI low 95 CI high
## [1,] 0.5412815 0.3945068  0.7426633
## attr(,"class")
## [1] "mipool"
```

Diarrhea

```r
pool_odds_ratio(with(dat_imp, expr=odds_ratio(vaccine_cov ~ diarrhea)))
```

```
##      pooled OR 95 CI low 95 CI high
## [1,] 0.9370858 0.5794052   1.515571
## attr(,"class")
## [1] "mipool"
```

Vomit

```
pool_odds_ratio(with(dat_imp, expr=odds_ratio(vaccine_cov ~ vomit)))
```

```
##      pooled OR 95 CI low 95 CI high
## [1,] 0.8352794 0.5083313   1.372514
## attr(,"class")
## [1] "mipool"
```

Abdominal pain

```
pool_odds_ratio(with(dat_imp, expr=odds_ratio(vaccine_cov ~ abd_pain)))
```

```
##      pooled OR 95 CI low 95 CI high
## [1,]  1.169572 0.7296263   1.874794
## attr(,"class")
## [1] "mipool"
```

Fatigue

```
pool_odds_ratio(with(dat_imp, expr=odds_ratio(vaccine_cov ~ fatigue)))
```

```
##      pooled OR 95 CI low 95 CI high
## [1,] 0.9631591 0.6867503   1.350819
## attr(,"class")
## [1] "mipool"
```

Loss of smell

```
pool_odds_ratio(with(dat_imp, expr=odds_ratio(vaccine_cov ~ olfac_loss)))
```

```
##      pooled OR 95 CI low 95 CI high
## [1,]  1.137034 0.7773557   1.663132
## attr(,"class")
## [1] "mipool"
```

Loss of taste

```
pool_odds_ratio(with(dat_imp, expr=odds_ratio(vaccine_cov ~ loss_taste)))
```

```
##      pooled OR 95 CI low 95 CI high
## [1,]  1.181084 0.8003242   1.742992
## attr(,"class")
## [1] "mipool"
```

Any respiratory symptom

```
for (i in 1:imputed_Data$m){
df <- dat_imp[[i]] %>%
  select(dyspnea,fatigue,desaturation,resp_disc)

soma <- function(x){
  if (sum(is.na(x))==4)
    return(NA_character_)
  else
    return(sum(!is.na(x) & x=="yes"))
}
dat_imp[[i]]$qt_sintomas_resp_aux <- apply(df,1,soma)

dat_imp[[i]] <-  dat_imp[[i]] %>%
  mutate(resp_symp = case_when(qt_sintomas_resp_aux >=1 ~ "yes",
                               qt_sintomas_resp_aux ==0 ~ "no",
                               TRUE ~ NA_character_))
}

pool_odds_ratio(with(dat_imp, expr=odds_ratio(vaccine_cov ~ resp_symp)))
```

```
##      pooled OR 95 CI low 95 CI high
## [1,] 0.5210252 0.3724448  0.7288791
## attr(,"class")
## [1] "mipool"
```

Any symptom

```
for (i in 1:imputed_Data$m){
df <- dat_imp[[i]] %>%
  select(dyspnea,fatigue,desaturation,resp_disc,
         fever,cough,sore_throat,diarrhea,vomit,abd_pain,olfac_loss,loss_taste)
soma <- function(x){
  if (sum(is.na(x))==12)
    return(NA_character_)
  else
    return(sum(!is.na(x) & x=="yes"))
}
dat_imp[[i]]$qt_sintomas_aux <- apply(df,1,soma)

dat_imp[[i]] <-  dat_imp[[i]] %>%
  mutate(symptom = case_when(qt_sintomas_aux >= 1 ~ "yes",
                             qt_sintomas_aux == 0 ~ "no",
                             TRUE ~ NA_character_))
}

pool_odds_ratio(with(dat_imp, expr=odds_ratio(vaccine_cov ~ symptom)))
```

```
##      pooled OR 95 CI low 95 CI high
```

```
## [1,] 0.4706185 0.2644071  0.8376544
## attr(,"class")
## [1] "mipool"
```

Characteristics of COVID-19 symptoms by vaccination status

ICU

```r
pool_odds_ratio(with(dat_imp, expr=odds_ratio(vaccine_cov ~ icu)))
```

```
##      pooled OR 95 CI low 95 CI high
## [1,] 0.5361446 0.3789471  0.7585517
## attr(,"class")
## [1] "mipool"
```

Intubation

```r
pool_odds_ratio(with(dat_imp, expr=odds_ratio(vaccine_cov ~ intubation)))
```

```
##      pooled OR 95 CI low 95 CI high
## [1,] 0.2267073 0.1160312  0.4429518
## attr(,"class")
## [1] "mipool"
```

Death

```r
pool_odds_ratio(with(dat_imp, expr=odds_ratio(vaccine_cov ~ death)))
```

```
##      pooled OR  95 CI low 95 CI high
## [1,] 0.1883021 0.08279405  0.4282638
## attr(,"class")
## [1] "mipool"
```
